# Characterization of Distinct Biofilm Cell Subpopulations and Implications in Quorum Sensing and Antibiotic Resistance

**DOI:** 10.1128/mbio.00191-22

**Published:** 2022-06-13

**Authors:** Taylor A. Dodson, Eric A. Carlson, Nathan C. Wamer, Chase N. Morse, Jennifer N. Gadient, Erin G. Prestwich

**Affiliations:** a Department of Medicinal and Biological Chemistry, University of Toledo, Toledo, Ohio, USA; b The College of Natural Sciences and Mathematics, NSM Instrumentation Center, University of Toledo, Toledo, Ohio, USA; University of South Florida; University of Pittsburgh School of Medicine

**Keywords:** *Pseudomonas aeruginosa*, antibiotic tolerance, biochemistry, biofilms, mass spectrometry, morphological variation, phenotypic variation, quorum sensing

## Abstract

Bacteria change phenotypically in response to their environment. Free swimming cells transition to biofilm communities that promote cellular cooperativity and resistance to stressors and antibiotics. We uncovered three subpopulations of cells with diverse phenotypes from a single-species Pseudomonas aeruginosa PA14 biofilm, and used a series of steps to isolate, characterize, and map these cell subpopulations in a biofilm. The subpopulations were distinguishable by size and morphology using dynamic light scattering (DLS) and scanning electron microscopy (SEM). Additionally, growth and dispersal of biofilms originating from each cell subpopulation exhibited contrasting responses to antibiotic challenge. Cell subpopulation surface charges were distinctly different, which led us to examine the ionizable surface molecules associated with each subpopulation using mass spectrometry. Matrix assisted laser desorption ionization time-of-flight (MALDI-TOF) mass spectrometry analysis of cell subpopulations revealed ions unique to each subpopulation of cells that significantly co-localized with ions associated with quorum sensing. Transcript levels of *algR*, *lasR*, and *rhlI* in subpopulations isolated from biofilms differed from levels in planktonic stationary and mid-log cell subpopulations. These studies provide insight into diverse phenotypes, morphologies, and biochemistries of PA14 cell subpopulations for potential applications in combating bacterial pathogenesis, with medical, industrial, and environmental complications.

## INTRODUCTION

All living cells dynamically express characteristic identities and behaviors. Though cellular differentiation has been methodically characterized in multicellular organisms, considerably less is known about the diversification of unicellular organisms. However, unicellular organisms can exhibit distinguishable phenotypes. For example, early work utilized stalking *Caulobacter* and sporulating *Bacillus* as models to describe bacterial morphogenic differentiation (1, 2). Subsequently, it was discovered that genetically similar bacteria can form physiologically distinct subpopulations (3) that appear to be regulated by chemical gradients (4). Recently, phenotypically distinct subpopulations originating from a P. aeruginosa PAO1 rugose small colony variant (RSCV) strain were visualized by scanning-transmission electron microscopy, leading to the isolation of two subpopulations with differential metabolic capacities and structural extracellular DNA abundance (5).

Biofilms are heterogeneous communities of cells surrounded by a matrix of extracellular DNA, exopolysaccharides, and proteins (6–8). This self-produced structural matrix aids in protecting cells from environmental stressors, including nutrient limitation, antibiotic treatment, and interspecies competition (9, 10). Once believed to originate from cell lysis (11), extracellular matrix formation is now thought to be a controlled process (12). Small molecules such as bis-(3′,5′)-cyclic dimeric GMP (cyclic-di-GMP), quorum sensing molecules, and small RNAs regulate the formation of biomass (12, 13).

Biofilm dwelling bacteria utilize small molecules to promote activities beneficial to the community (14, 15) and can lead to phenotypically distinct cells in a single species biofilm (16). Cyclic-di-GMP is linked to pro-biofilm traits such as swarming, surface adhesion production, and antibiotic tolerance (17). Additionally, quorum sensing systems regulate the production and distribution of small molecules that initiate biofilm phenotypes (18, 19). Previously, MALDI-MS imaging (20), confocal Raman microscopy (21), and 3D OrbiSIMS (22) have been used to study heterogeneity and small molecule signaling in biofilms. However, it remains unclear whether specific molecules are associated with particular cells in biofilms.

P. aeruginosa forms prolific biofilms in both environmental and medical settings, prompting its use as a model system for biofilm growth and treatment (23), and the evolution of physiologically distinct cell subpopulations during biofilm development (5, 24). Cells can experience nutrient gradients or small-molecule signals depending on their localization within biofilms, subsequently affecting physiological changes and biomass production (4). The World Health Organization (WHO) lists P. aeruginosa as a critical priority pathogen (25), highlighting the importance of understanding its pathogenicity and life cycle. Its biofilm forming ability aids in antibiotic tolerance (23), though the cellular and environmental heterogeneity in a biofilm could also impact antibiotic sensitivity. The cellular transition from planktonic to sessile is thought to result from extracellular and intracellular small molecule signaling, though these processes are still being elucidated (10).

Understanding bacterial heterogeneity is essential to diagram pathways responsible for biofilm accumulation and pathogenicity. Here, three morphologically distinct cell subpopulations in a single-species PA14 biofilm were identified via SEM, isolated utilizing a combination of enzymatic digestion and centrifugation, and phenotypically characterized. This report describes the mapping of distinct bacterial cell subpopulations in a single species biofilm via MALDI imaging, allowing cell subpopulations to be visualized directly through their endogenous properties. Our findings advance current knowledge of biofilm heterogeneity enabling future studies of bacterial ecology and pathogenesis.

## RESULTS

### Identification of three PA14 biofilm cell subpopulations.

**FIG 1:**
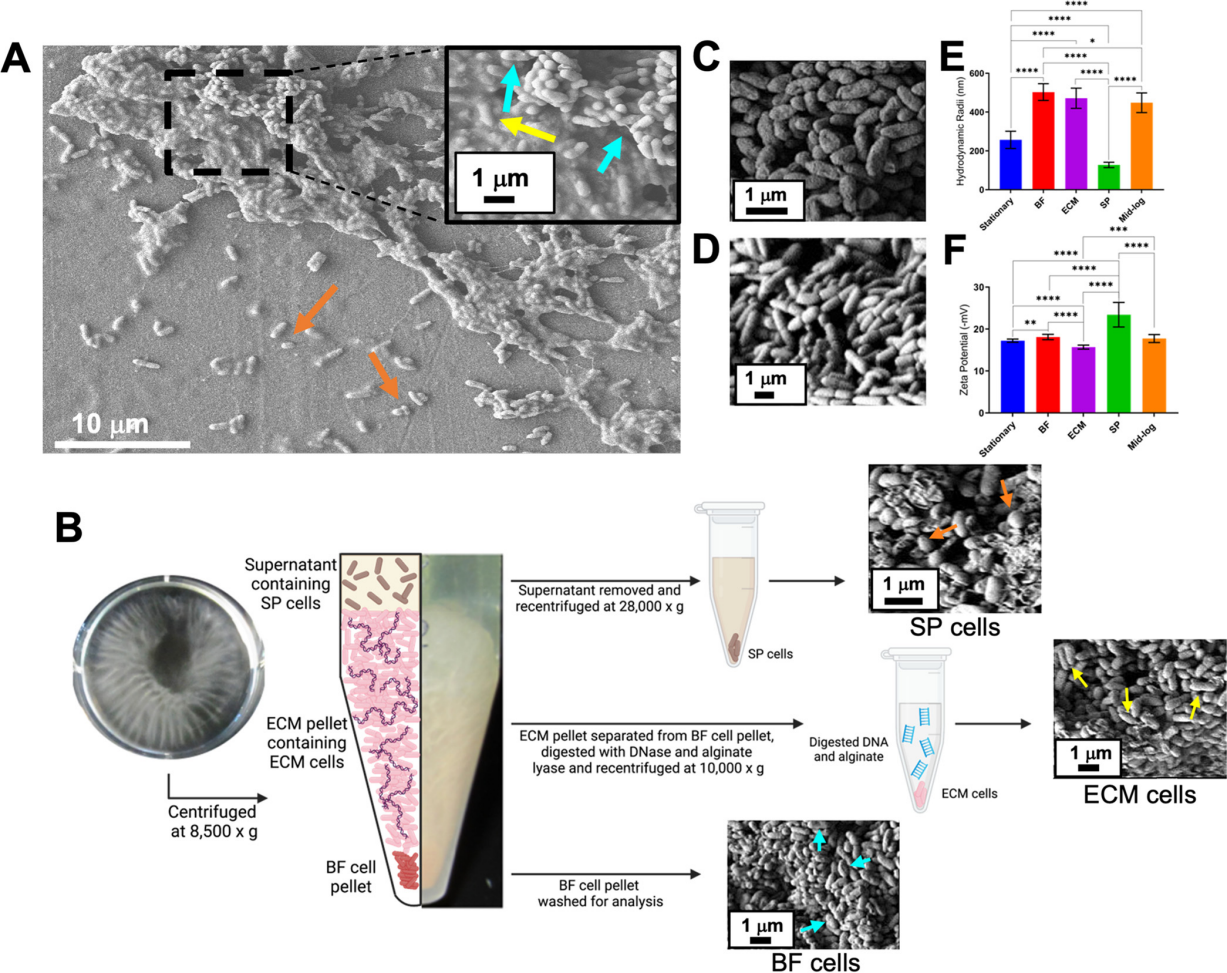
SEM, DLS particle size analysis, and zeta potential measurements of PA14 cell subpopulations. A. A PA14 biofilm was imaged via SEM. The area represented by the dashed box was magnified to show biofilm (BF) and extracellular matrix (ECM) cell morphology and matrix structure. The yellow arrow highlights the ECM cells encased in a gelatinous matrix, while the blue arrows indicate dented BF cells and the associated matrix. Coccobacillus morphology in supernatant (SP) cells is highlighted by orange arrows. B. Optical images of stationary biofilm growth (picture taken from above) and resulting biofilm layers after centrifugation (side view). SEM images are of biofilm cells separated via centrifugation. Shown are three layers of biofilm, the SP, ECM, and BF pellet. SP cells were imaged via SEM following overnight centrifugation, and orange arrows point at coccobacillus morphology. ECM cells were imaged after enzymatic digestion of the extracellular matrix and further centrifugation, and bacilli morphologies are highlighted with yellow arrows. Blue arrows point out kinked/dented bacilli morphology of BF cells. C. SEM image of stationary-phase planktonic cells. D. SEM image of mid-log-phase planktonic cells. E. Average hydrodynamic radii from DLS particle size analysis. Significance was determined using the unpaired two-tailed *t* test to compare each subpopulation; stationary-phase planktonic cells (256.78 nm ± 44.48, *n* = 12), BF cells (502.90 nm ± 43.14, *n* = 10), ECM cells (471.14 nm ± 52.05, *n* = 33), SP cells (127.52 nm ± 13.65, *n* = 9), and mid-log-phase cells (448.20 nm ± 50.73, *n* = 8). Data are mean of biological replicates (n) ± SD, ****, *P* < 0.0001; ***, *P* < 0.001; **, *P* < 0.01; *, *P* < 0.05. F. Average cell surface charge from zeta potential analysis (^−^mV). Significance was determined using the unpaired two-tailed *t* test to compare each subpopulation; stationary-phase planktonic cells (^−^17.22 ± 0.84 mV, *n* = 21), mid-log-phase cells (^−^17.74 ± 0.95 mV, *n* = 8), BF cells (^−^18.10 ± 0.63 mV, *n* = 11), ECM cells (^−^15.70 ± 0.48 mV, *n* = 11), and SP cells (^−^23.42 ± 2.93 mV, *n* = 12). Data are mean of biological replicates (n) ± SD, ****, *P* < 0.0001; ***, *P* < 0.001; **, *P* < 0.01; *, *P* < 0.05.

To study bacterial biofilms, P. aeruginosa PA14 was chosen as a model organism. SEM was performed with a single-species PA14 biofilm, and three potential cellular morphologies were visualized having different shapes, sizes, or matrix features (Fig. 1A). Previous publications described the influence of P. aeruginosa subpopulations toward the biofilm environment, including production of extracellular matrix (5, 26) and antibiotic tolerance (24, 27), thus establishing distinct roles of cell subpopulations. Hence, a need for characterization of P. aeruginosa biofilm cell subpopulations was defined. Here, bacillus and coccobacillus cells were identified with no visible encompassing matrix, contrasting with two other bacillus cell morphologies with visible matrix. The matrix associated with one cell morphology allowed visibility of dented cell surface features, while the other matrix was thick which prevented visualization of cellular surface features (Fig. 1A).

These morphologically distinct subpopulations were isolated for quantitative characterization. Biofilms were grown as detailed in the Materials and Methods, and the whole culture was centrifuged for subpopulation harvesting. After centrifugation, three layers were visible: (i) cell pellet, (ii) gelatinous matrix layer, and (iii) liquid supernatant (Fig. 1B). Each of these layers contained cells, thus suggesting the efficient separation of three cell subpopulations. The frequency of each cell subpopulation in the biofilm was calculated and reported in Table S1. Stationary (Fig. 1C) and mid-log (Fig. 1D) phase planktonic cells were also visualized by SEM.

10.1128/mbio.00191-22.1 TABLE S1 Comparison of the zeta potential, biomass accumulation, CFU/mL, and cell frequencies between subpopulations. Download Table S1, DOCX file, 0.02 MB. Copyright © 2022 Dodson et al. 2022 Dodson et al. https://creativecommons.org/licenses/by/4.0/ This content is distributed under the terms of the Creative Commons Attribution 4.0 International license.

The cell pellet and gelatinous matrix layers from initial centrifugation of the whole biofilm (Fig. 1B) were isolated. The cell pellet contained biofilm (BF) cells, which exhibited dented and kinked bacilli morphologies (Fig. 1B). To confirm if the dented and kinked bacilli morphology of BF cells was an artifact of SEM preparation or analysis under vacuum, brightfield and fluorescence microscopy was performed (Fig. S1A). Some BF cells appeared as curved and vibrio-like. This vibrio-like shape is likely representative of the characteristic BF cell dents and kinks as seen via SEM analysis. The cells from the gelatinous matrix layer (ECM cells) required an enzymatic digest to liberate the cells. It is well defined that a main structural component of P. aeruginosa biofilm matrices is extracellular DNA (5, 28). However, treating the gelatinous matrix pellet with DNase alone did not free ECM cells from the matrix. Alginate is reported as a component of P. aeruginosa biofilm matrices (29, 30). Thus, addition of alginate lyase, an enzyme that cleaves glycosidic bonds in polysaccharides by β-elimination (31), with DNase efficiently liberated the ECM cells from the gelatinous matrix (Fig. 1B). This enabled phenotypic and morphologic analysis of ECM cells. ECM cells were primarily bacilli morphology (Fig. 1B).

10.1128/mbio.00191-22.2 FIG S1 Control experiments to show the effects of biofilm cell subpopulation harvesting conditions, cell phenotype, and cell viability. Cultured biofilm subpopulations and RSCV colony morphology. A. Representative brightfield and fluorescence microscopy (live/dead staining) depicting morphologies and viability of stationary planktonic cells, biofilm (BF) cells, extracellular matrix (ECM) cells, and supernatant (SP) cells. Blue arrows indicate kinked/dented bacilli cell morphology. Yellow arrows indicate bacilli morphology characteristic of ECM cells. Orange arrows indicate coccobacillus cell morphology of live SP cells. B. Growth curves of stationary planktonic phase cells, and SP cells isolated after centrifuge time points of 3 h or overnight. SP cells that were analyzed after centrifugation for 3 h or overnight showed similar growth patterns, with a longer lag phase than stationary planktonic phase cells. C. Bar graph showing the comparison of the average radii of SP cells (in nm) measured by dynamic light scattering (DLS). Significance was determined using the unpaired two-tailed t-test, and SP cells isolated by 3-hour centrifugation (*n* = 3, *P* value 0.0273, t = 2.582, df = 10), SP cells isolated by 6-hour centrifugation (*n* = 2, *P* value 0.2037, t = 1.37, df = 9), and SP cells isolated by 3- and 6-hour centrifugation averaged (*n* = 5, *P* value 0.4069, t = 0.8596, df = 12) were compared to SP cells isolated by overnight centrifugation (*n* = 9). Sample sizes are biological replicates (n). D. Representative SEM images taken of SP cells after 3 or 6 h of centrifugation, demonstrating similar cellular morphologies regardless of centrifugation time. Orange arrows point toward coccobacillus cell morphology. E. Stationary planktonic cell viability studies with centrifugation parameters which mimic biofilm harvesting time and centrifugation speed. Time 45 minutes: cells were centrifuged for 45 minutes at 11,000 × *g* (BF cell harvest conditions) (*n* = 3, *P* value = 0.0767). Overnight: cells were centrifuged overnight at 28,000 × *g* (SP cell harvest conditions) (*n* = 3, *P* value = 0.0728). 45 minutes versus overnight: (*n* = 3, *P* value = 0.4442). F. Zeta potentials of BF and stationary-phase planktonic cells after enzymatic digestion used to liberate ECM cells from extracellular matrix. Stationary phase planktonic cell surface charge (*n* = 4) was not significantly different from normal sample preparation (*P* = 0.0874). BF cell surface charge (*n* = 4) was significantly different from normal sample preparation (****, *P* < 0.0001). ECM and BF cell surface charge were not significantly different after both were respectively digested with the ECM enzymatic digestion (*P* = 0.2420). Significance was determined using the unpaired two-tailed t-test and (n) are biological replicates. G. Representative image of colonies grown from isolated SP cells on an LB agar plate to show visible size difference of normal colonies and RSCV colonies. Yellow arrows point at RSCV colonies. Blue arrows point at SP colonies. H. Light microscope image of colonies grown from harvested cells. The yellow arrows point at RSCV colonies in the biofilm-associated cell types (BF, ECM, and SP). Download FIG S1, TIF file, 1.5 MB. Copyright © 2022 Dodson et al. 2022 Dodson et al. https://creativecommons.org/licenses/by/4.0/ This content is distributed under the terms of the Creative Commons Attribution 4.0 International license.

The liquid supernatant contained cells after initial centrifugation, termed supernatant (SP) cells, and were isolated from the liquid supernatant with further centrifugation (Fig. 1B). SP cells were coccobacillus and wrinkled (Fig. 1B). To confirm morphology and viability of SP cells, live/dead staining with fluorescence microscopy analysis was performed (Fig. S1A). There were both live and dead coccobacillus SP cells. Different centrifugation times were tested (3 h, 6 h, or overnight) for collection of all viable SP cells from the supernatant. Differing centrifugation times pelleted cells with similar properties (Table S1 and Fig. S1B to S1D). Additionally, cell viability was assessed to determine the effects of centrifugation parameters (time and speed). The results demonstrated unchanged cell viability due to centrifugation (Fig. S1E). Thus, to maximize cell recovery, overnight centrifugation was utilized to isolate SP cells for all experiments unless stated otherwise.

The isolated SP, ECM, and BF cells were analyzed to assess cell surface features. Cell size was quantified by DLS. BF, ECM, and planktonic mid-log cells were similarly sized, however, stationary-phase planktonic and SP cells were significantly smaller (Fig. 1E). Cell membrane potentials (zeta potentials) were analyzed, and SP cells exhibited the lowest surface charge (most negative) whereas ECM cells exhibited the highest surface charge (least negative) (Fig. 1F). As a control to test if the less negative ECM cell surface charge was an artifact of enzymatic treatment, planktonic and BF cells were treated with the same enzymatic digest. After enzymatic treatment, the zeta potential of planktonic cells was unaffected, though the BF cell zeta potential was equal to that of ECM cells (Fig. S1F). This suggests that the enzymatic digest removes cell surface matrix and thus is responsible for the higher zeta potential observed in ECM cells.

### Cell subpopulations exhibit differential growth patterns and phenotypes.

**FIG 2:**
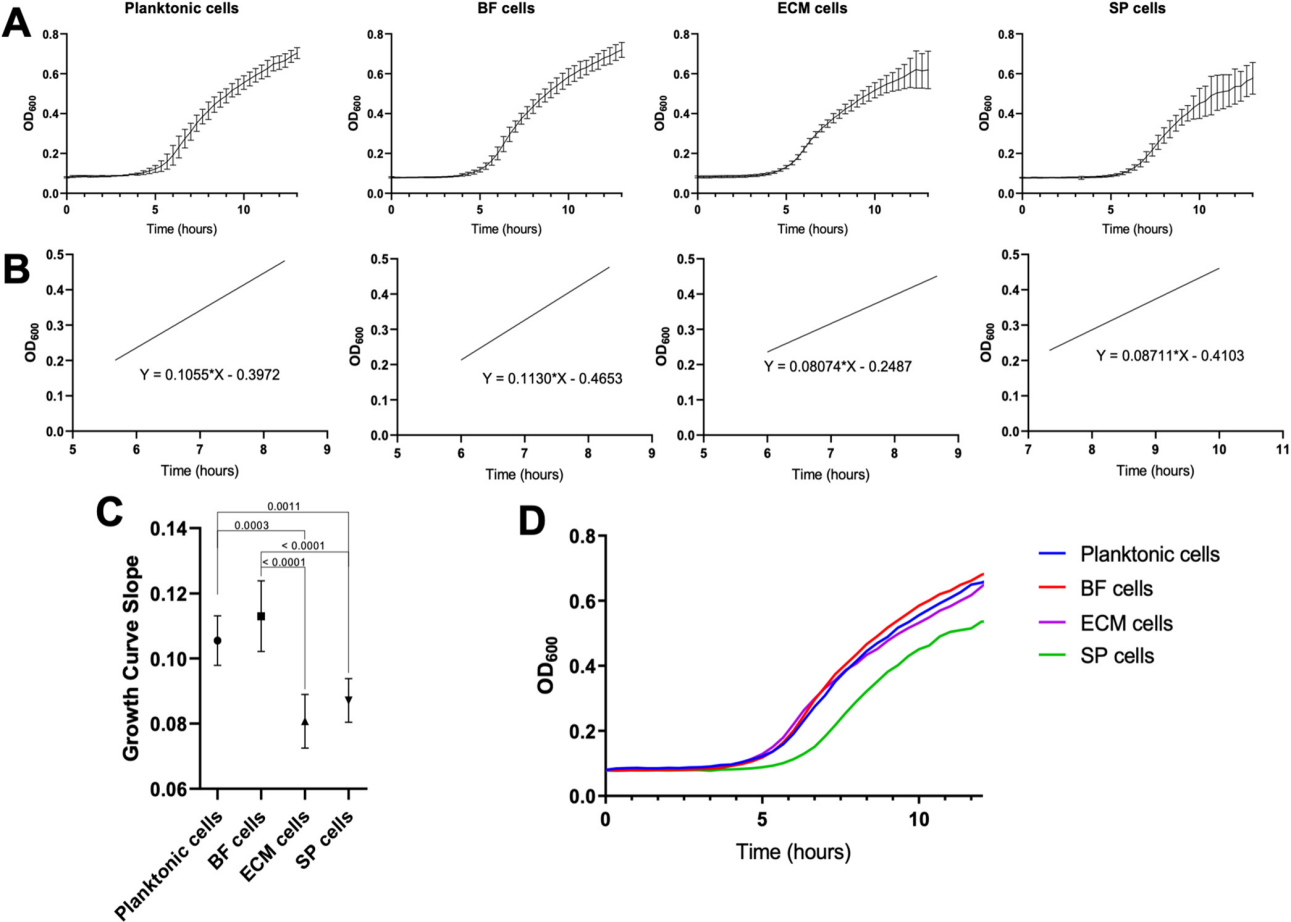
Cell subpopulation growth analysis. A. Averaged growth curves for stationary planktonic (*n* = 4), BF (*n* = 9), ECM (*n* = 9), and SP (*n* = 9) subpopulations. Four technical replicates per biological replicate (n). B. Cell exponential growth was fit to linear regression curves. Graphs depict averaged growth curve exponential phases from each cell type, which shows differential exponential growth. Exponential phases were fit to linear curves by using OD_600_ values from 0.20 ± 0.01 to 0.45 ± 0.01 (R^2^ > 0.985). Linear regression curves of averaged data: stationary-phase planktonic cells y = 0.1055x - 0.3972, BF cells y = 0.113x - 0.4653, ECM cells y = 0.08074x - 0.2487, and SP cells y = 0.08711x - 0.4103. C. Slope comparison between the linear regression curves for the stationary planktonic (0.1055 ± 0.0076), BF (0.113 ± 0.0108), ECM (0.08074 ± 0.0082) and SP (0.08711 ± 0.0067) subpopulations. Students *unpaired t test* for statistical analysis, graph depicting *P* value comparisons to the 95% confidence interval. D. Averaged growth curves of each subpopulation depicting longer lag time for the SP subpopulation compared to other subpopulations.

Since bacterial cell structure is associated with cellular function (32, 33), it was hypothesized that differential cell morphologies (shape, size, surface charge) correlate with other cellular characteristics like viability and proliferation. Stationary planktonic, BF, and ECM cells all exhibited similar viability, but SP cells demonstrated an order of magnitude lower viability (Table S1). However, because longer centrifugation times at a higher speed did not affect cell viability (Fig. S1E) it is likely that nonviable cells also pelleted with overnight centrifugation. Cell proliferation was examined by monitoring the regrowth of harvested cells (growth curves). All cell subpopulations were proliferative but growth patterns between the subpopulations differed (Fig. 2A). Exponential growth phases were fit to linear regression curves for slope comparisons (Fig. 2B). BF and stationary planktonic phase cell linear regressions were significantly different from SP and ECM cell linear regression curves (Fig. 2C). Stationary planktonic phase, BF, and ECM cells stayed in lag phase for about 3 h, while SP cells spent approximately 5 h in lag phase (Fig. 2D) irrespective of the centrifugation time used to isolate the SP cells (Fig. S1B) or the number of viable cells initially inoculated (Table S1). This suggests that SP cells could be in a state of dormancy while in biofilms, and that they can resuscitate when regrown planktonically (4).

**FIG 3:**
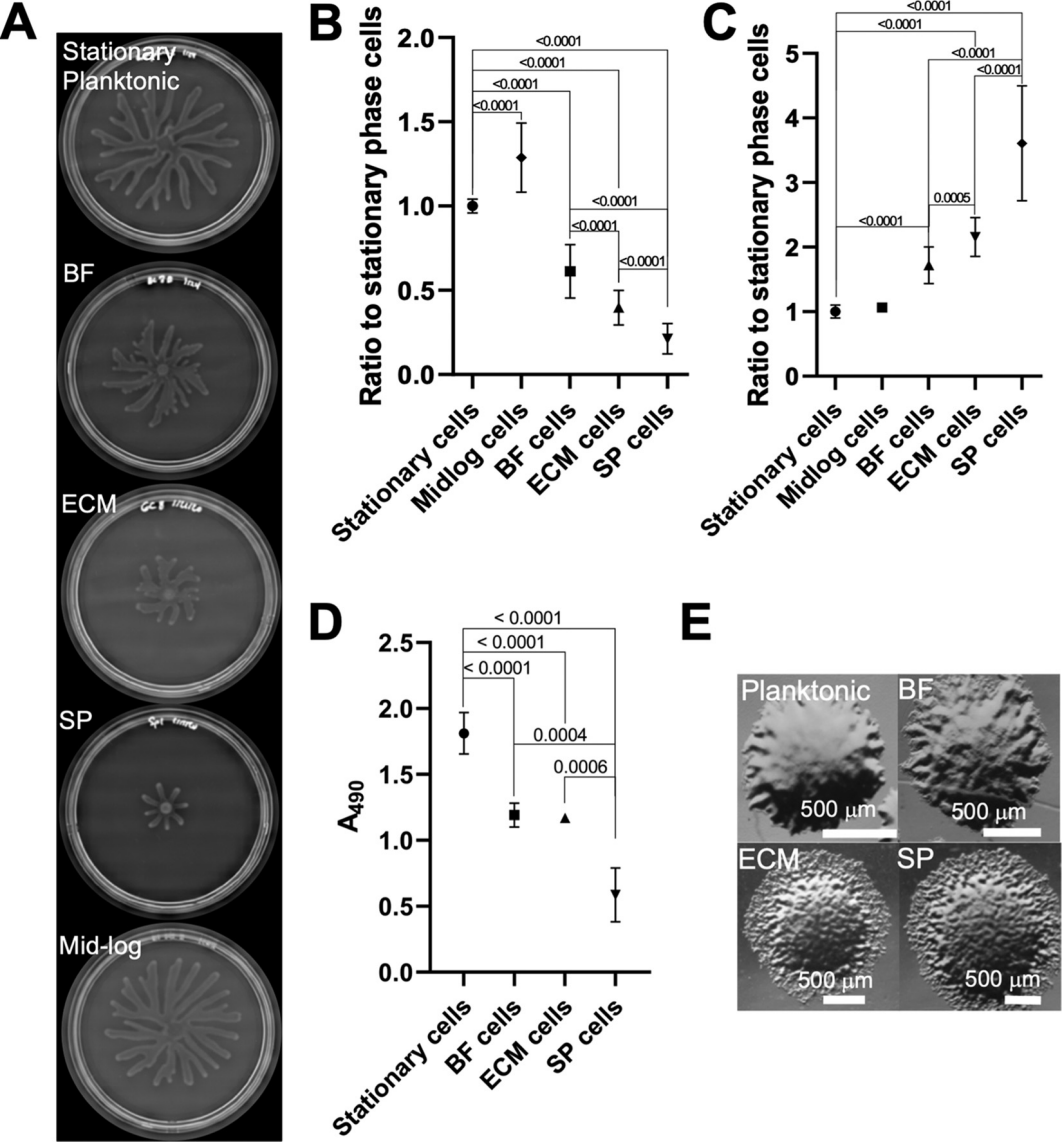
Phenotypic analyses of biofilm related behaviors in PA14 cell subpopulations. A. Representative images of swarming on semisolid agar, each incubated for 24 h. B. Average quantified area of swarming of each cell subpopulation, calculated using Image J. Data shown as ratios between the subpopulation average swarming areas and average swarming area of stationary-phase planktonic cells. Significance was determined using the unpaired two-tailed *t* test. Averages and standard deviations calculated from biological replicates (n) (1.00 AU ± 0.04, stationary-phase planktonic cells, *n* = 18) (0.612 AU ± 0.158, BF cells, *n* = 18), (0.397 AU ± 0.102, ECM cells, *n* = 16), (0.212 AU ± 0.0907, SP cells, *n* = 18), (1.287 AU ± 0.204, mid-log-phase cells, *n* = 15). Mid-log swarming versus BF, ECM, or SP cell swarming have a *P* < 0.0001. C. Static biomass accumulation stained with crystal violet (CV). Data represented relative to stationary-phase planktonic biomass. Significance was determined using the unpaired two-tailed *t* test. Standard deviations calculated from biological replicates as specified in Table S1. (stationary-phase planktonic cells, *n* = 12), (BF cells, *n* = 12), (ECM cells, *n* = 17), (SP cells, *n* = 12). Mid-log swarming versus BF, ECM, or SP cell swarming have a *P* < 0.0001. D. Quantification and comparison of exopolysaccharide production by each cell subpopulation using the Congo red assay without accounting for differential cellular growth. Data represented as the 490 nm averages and standard deviation calculated from biological replicates (n): stationary-phase planktonic cells, *n* = 22; BF cells, *n* = 6; ECM cells, *n* = 5; SP cells, *n* = 4. Significance was determined using the unpaired two-tailed *t* test, with *P*-values labeled on the graph. E. Colony morphology of stationary-phase planktonic cells, BF cells, ECM cells, and SP cells when plated on LB agar.

Polysaccharide development, biomass production, and cell motility were assessed in each cell subpopulation as these phenotypes are related to biofilm development in P. aeruginosa (15). These biofilm-related phenotypes were measured through cell motility or dye-based assays (34–36). Quantification of cellular swarming motilities revealed significant variations between each of the different subpopulations (SP cells < ECM cells < BF cells < stationary-phase planktonic cells) (Fig. 3A and B). Swarming motilities and biomass accumulation are inversely related in P. aeruginosa (37). The biomass formed by the different isolated cell subpopulations was quantified using a crystal violet assay (36) and cellular subpopulation abilities to form biomass were inversely related to cell motility: planktonic cells < BF cells < ECM cells < SP cells (Fig. 3C). Exopolysaccharides are important constituents of P. aeruginosa biofilm extracellular matrices (38, 39). To quantify exopolysaccharides, such as Pel, produced by each cell subpopulation, a Congo red assay was utilized (35). Stationary planktonic cells produced less exopolysaccharides than other cell subpopulations and SP cells produced the most exopolysaccharides (Fig. 3D).

Colony morphology has been used to identify genes associated with biofilm production, as wrinkled colonies indicate higher biofilm formation (40). Thus, the colonies produced when each cell subpopulation was plated on LB agar were screened and colonies derived from stationary planktonic phase cells were smooth. However, BF cells produced wrinkled colonies and ECM and SP cell colonies were hyper-wrinkled (Fig. 3E). These observations are consistent with the higher biomass production seen in BF, ECM, and SP subpopulations (Fig. 3C). Additionally, when plated, roughly 10% of BF and ECM and 20% of SP cell colonies were RSCVs (Table S1 and Fig. S1G and S1H).

The different phenotypes observed between freshly harvested cell subpopulations were compared to phenotypes observed from cell subpopulations re-cultured in liquid media and grown to exponential phase (OD_600_ 0.3) to determine if phenotypes were transient. After growth to exponential phase, the cellular phenotypes were assessed and SP, BF, and ECM cell surface charges, biomass accumulation, and swarming motilities of each subpopulation were insignificantly different from planktonic cell measurements (Fig. S2A to S2E). The observed BF, ECM, and SP cell phenotype differences from freshly harvested cells are transient characteristics, and growth conditions in a sessile biofilm are necessary to produce these variations. Thus, it is likely that the phenotypes examined here are not caused by genetic mutations but are a result of small-molecule signaling or epigenetic changes (41) that can affect genetic regulation.

10.1128/mbio.00191-22.3 FIG S2 Phenotypic and antibiotic assays performed with isolated cells. Cell subpopulation phenotypic assay analyses show BF, ECM, and SP subpopulations revert to planktonic phase cell subpopulation phenotypes. Unpaired t-tests were performed to determine confidence intervals at 95% compared to planktonic stationary-phase cells (except where noted). A. Zeta potentials of BF, ECM, and SP cell subpopulations regrown to mid-log planktonic phase. Insignificant differences: (BF cells *P* = 0.312, *n* = 5, t = 1.033, df = 24), (ECM cells *P* = 0.7959, *n* = 6, t = 0.2615, df = 25), (SP cells *P* = 0.3917, *n* = 3, t = 0.8738, df = 22). B. DLS particle size analysis of BF, ECM, and SP cell subpopulations regrown to mid-log planktonic phase. Insignificant differences: (BF cells *P* = 0.2736, *n* = 5, t = 1.14, df = 14), (ECM cells *P* = 0.5885, *n* = 4, t = 0.5538, df = 14), (SP cells *P* = 0.2218, *n* = 4, t = 0.5538, df = 14) C. Biomass accumulation of BF, ECM, and SP cell subpopulations re-cultured and normalized to planktonic stationary-phase cell biomass accumulation (CV assay). ECM compared to planktonic mid-log-phase cells. Insignificant differences: (BF cells *P* = 0.8502, *n* = 3, 0.2014, df = 4), (ECM cells *P* = 0.0672, *n* = 3, t = 2.053, df = 10), (SP cells *P* = 0.2489, *n* = 3, t = 1.348, df = 4) D. Swarming areas of BF, ECM, and SP cell subpopulations re-cultured and measured areas normalized to planktonic stationary-phase cells. Significance determined compared to planktonic mid-log-phase cells. Insignificant differences: (BF cells *P* = 0.6006 *n* = 3, t = 0.5342, df = 16), (ECM cells *P* = 0.4100, *n* = 3, t = 0.846, df = 16), (SP cells *P* = 0.4509, *n* = 3, t = 0.7729, df = 16) E. Crystal violet assay depicting biomass accumulation after re-culturing. Isolated cells from harvested biofilms were grown on LB agar and colonies were re-cultured in LB broth. Assay performed and analyzed as mentioned in methods. No significant differences in biomass accumulation were seen in re-cultured cells compared to planktonic cell biomass accumulation. Unpaired two-tailed t-test: BF *P* = 0.8502 (*n* = 3), ECM *P* = 0.0672 (*n* = 3), SP *P* = 0.2489 (*n* = 3). Sample size from each analysis were biological replicates (n), with four technical replicate per biological replicate for the biomass accumulation assay. F. Accumulation and dispersion assays comparing stationary planktonic cell tobramycin or colistin susceptibilities against the susceptibilities of stationary planktonic cells treated with the ECM digest or centrifuged at 28,000 × *g* (to mimic SP cell harvesting). Data are an average of: % biomass accumulation, normalized to % biomass accumulation of untreated cells; or % biomass remaining after treatment with colistin or tobramycin, normalized to % biomass remaining of untreated cells. *n* = 3 biological replicates. Two-way ANOVA with *post hoc* Tukey’s tests to correct for multiple comparisons were performed and showed no significant differences between any cell antibiotic susceptibilities (*P* values > 0.05). Download FIG S2, TIF file, 2.0 MB. Copyright © 2022 Dodson et al. 2022 Dodson et al. https://creativecommons.org/licenses/by/4.0/ This content is distributed under the terms of the Creative Commons Attribution 4.0 International license.

**FIG 4:**
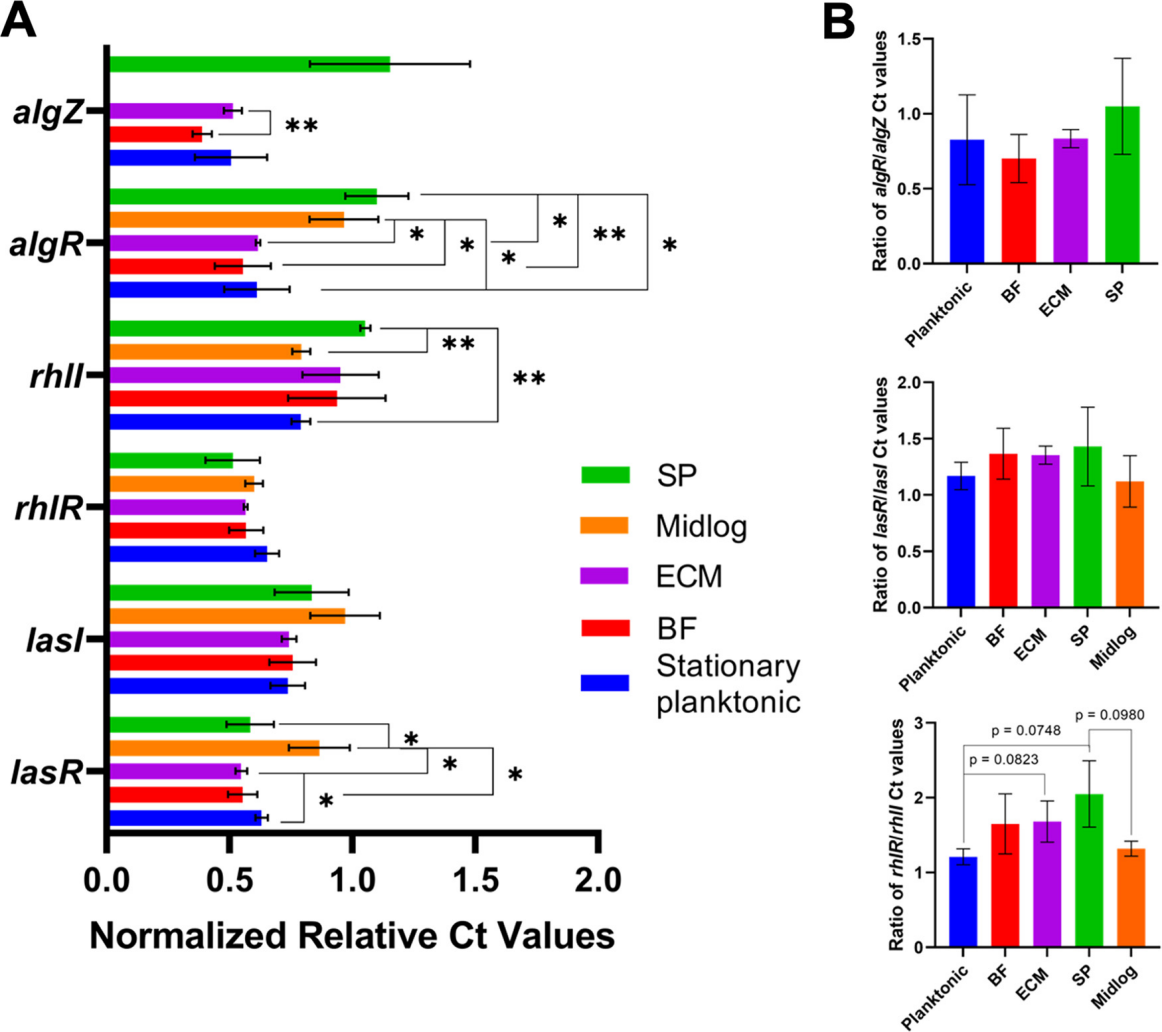
*algZ/algR*, *lasR/lasI*, and *rhlR/rhlI* expression levels in PA14 cell subpopulations analyzed via RT-qPCR. A. Average Ct values of each transcript normalized to expression (Ct values) of the reference transcript, *fabD*. Statistical significance calculated by *unpaired t test*. *, *P* < 0.05, **, *P* < 0.005, *n* ≥ 3. B. Ratios of normalized Ct values from A. The graph on the top shows the ratio of *algZ* and *algR*. The graph in the center shows the ratio of *lasI* and *lasR*. The graph on the bottom shows the ratio of *rhlI* and *rhlR*. Statistical significance calculated by unpaired two-tailed *t* tests.

To assess genetic regulation of quorum sensing and biomass production, transcript levels were measured. Biofilm-associated gene transcripts, *lasR*, *lasI*, *rhlR*, *rhlI*, (quorum sensing) (19) *algR*, and *algZ* (biomass/alginate production) (19, 42) were quantified via reverse transcription quantitative PCR (RT-qPCR). The average cycle threshold (Ct) values of these transcripts, normalized to *fabD* (43, 44), are shown in Fig. 4A. Significantly higher transcript levels of *rhlI* in SP cells compared to planktonic cells, and significantly lower *lasR* transcript levels in BF, ECM, and SP cells than in mid-log cells were observed. Notably, *algZ* levels in mid-log cells were undetectable, though were significantly higher in BF cells versus ECM cells. Levels of *algR* were significantly higher in SP and mid-log subpopulations than the other cell subpopulations (Fig. 4A). Additionally, the ratio of *rhlR* to *rhlI* transcripts are different in ECM and SP cells compared to planktonic cells (Fig. 4B), suggesting that the balance of these transcripts vary in these cell subpopulations.

### Biofilm accumulation and surface dissociation in the presence of antibiotics differ between subpopulations.

**FIG 5:**
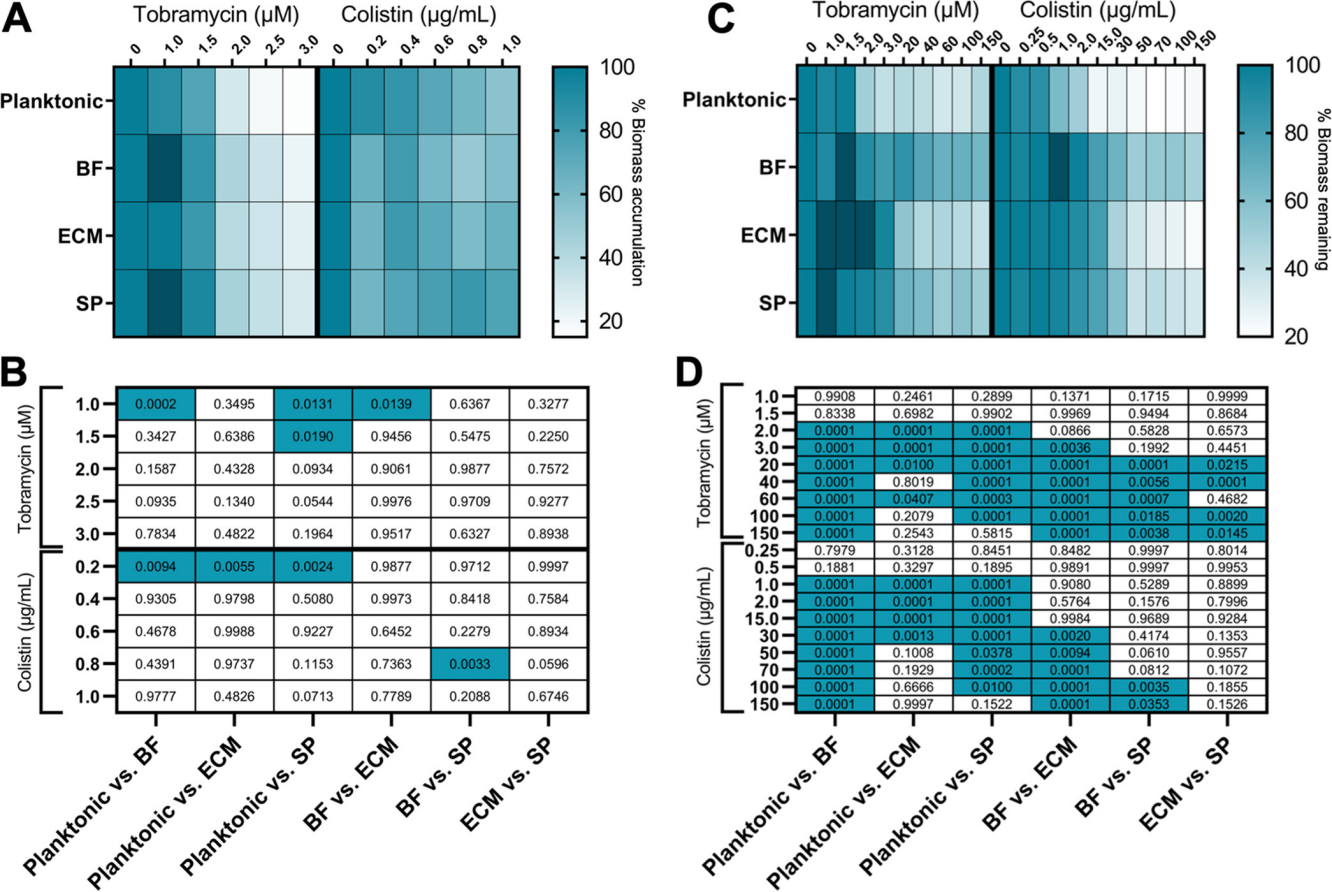
Antibiotic tolerance assays to test accumulation of biomass or dispersion of established biofilms with tobramycin or colistin treatment. A. Percent biomass accumulation by cell subpopulations incubated with media containing tobramycin or colistin. Resulting static biomass accumulation was measured by CV staining and absorbance measurement at OD_550_. Data are presented relative to biomass accumulation of the subpopulation without treatment. Statistics shown in Table S2. B. Statistical comparison of cell subpopulation biomass accumulation responses to tobramycin or colistin challenge. Two-way ANOVA with *post hoc* Tukey’s test was performed on average % biomass accumulation (Table S2) of cell subpopulations, where blue indicates *P* < 0.05 and white indicates *P* > 0.05, with numerical *P*-values shown. C. Percent biomass remaining after surface dissociation of mature biofilms challenged with various concentrations of tobramycin or colistin. After dissociation, remaining biomass was stained with CV and absorbance measured at OD_550._ Data are presented relative to the amount of biomass without treatment. Statistics shown in Table S2. D. Statistical comparison of cell subpopulation biomass dispersal responses to tobramycin or colistin challenge. Two-way ANOVA with *post hoc* Tukey’s test was performed on average % biomass remaining (Table S2) of cell subpopulations, where blue indicates *P* < 0.05 and white indicates *P* > 0.05, with numerical *P*-values shown.

P. aeruginosa biofilms are inherently antibiotic-tolerant (45), possibly as a consequence of phenotypically distinct cell subpopulations (27). To assess whether cell subpopulations respond differently to antibiotic treatment, tobramycin and colistin were delivered separately to each cell subpopulation in the presence (mature biofilm) or absence of biomass (before biofilm production). Tobramycin and colistin are antibiotics commonly used to treat P. aeruginosa infections (46, 47). Upon treatment with 1.0 μM tobramycin, BF cells produced significantly more biomass than ECM and planktonic cells, and SP cells produced significantly more biomass than planktonic cells. SP cells also produced more biomass than planktonic cells when challenged with 1.5 μM tobramycin. Interestingly, compared with planktonic cells, BF, ECM, and SP cells produced significantly less biomass when treated with 0.2 μg/mL colistin. Additionally, with 0.8 μg/mL colistin challenge, SP cells produced significantly more biomass than BF cells (Fig. 5A and B and Table S2). Mature biofilms grown from BF, ECM, and SP cell subpopulations were typically more tolerant to antibiotic treatment than biofilms grown from stationary planktonic cells (Fig. 5C). The biomasses grown by BF cells were the most antibiotic tolerant. Whereas those biomasses grown from planktonic cells were the most susceptible (Fig. 5C and D and Table S2). Eradication of any biomass did not occur with treatment by any concentration of antibiotic tested.

10.1128/mbio.00191-22.4 TABLE S2 Crystal violet antibiotic assays. Download Table S2, DOCX file, 0.02 MB. Copyright © 2022 Dodson et al. 2022 Dodson et al. https://creativecommons.org/licenses/by/4.0/ This content is distributed under the terms of the Creative Commons Attribution 4.0 International license.

The effects of cell isolation procedures to antibiotic susceptibility were assessed. Stationary planktonic cells were treated either with the enzymatic digest, to mimic ECM cell harvesting or centrifuged overnight, to mimic SP cell harvesting. The antibiotic susceptibilities were tested and compared to those of stationary planktonic cells harvested normally. Antibiotic susceptibility was unaffected by cell harvesting procedures (Fig. S2F).

### The detection of differentially expressed molecules associated with cell subpopulations can be utilized for characterization and identification in a complex biofilm.

Upon identifying surface charges characteristic of each subpopulation (Fig. 1F), different ionizable molecules were hypothesized to be associated with the surface of each cell subpopulation. To investigate the differences in cell surface molecules, each cell subpopulation was analyzed by MALDI-TOF (48) using gold as a matrix (49). Mass spectral profiles were obtained from isolated PA14 cell subpopulations and ions characteristic of each cell subpopulation were observed. Analysis revealed two unique ions specific to BF cells, four unique ions specific to ECM cells, and eight unique ions specific to SP cells (Table 1). Collision induced dissociation (CID) was performed on ions associated with each subpopulation to aid in identification (Table 1 and Fig. S3A to S3I, S4A to S4G, S5A to S5T).

**TABLE 1:** MALDI-TOF detected unique ions in each cell subpopulation and putative identification via MALDI-CID in positive ion mode

Unique ion	Compound	BF cells	ECM cells	SP cells
*m/z* 293	**Rha-C_7_**	X		
*m/z* 329	Rha-C_8_			X
*m/z* 335 (BF) *m/z* 379 (SP)	**Rha-C_10_**	X		X
*m/z* 383	Rha-C_12:1_			X
*m/z* 702	**Rha-Rha-C_10_-C_12_**			X
*m/z* 466	C_9:1_-2-alkyl-4-quinolone		X	
*m/z* 356	C_12:1_-2-alkyl-4-quinolone			X
*m/z* 394	**N-hexanoyl-l-homoserine lactone**		X	
*m/z* 404	Probable rhamnolipid			X
*m/z* 430	Unidentified		X	
*m/z* 463	Unidentified			X
*m/z* 482	Unidentified		X	
*m/z* 616	Unidentified			X

Bold text indicates ion was identified by CID compared to commercial standard.

*Rhamnolipid abbreviations explained elsewhere (50) and in Table S3.

10.1128/mbio.00191-22.5 FIG S3 Positive ion mode MALDI CID of BF cell ions. A. Spectrum of ion *m/z* 293 from BF cells. B. Spectrum of ion *m/z* 293 from R95 mono-rhamnolipid standard from AGAE Technologies. C. Observed fragmentation of Rha-C_7_ ion *m/z* 293 [M + H]^+^. D. Spectrum of ion *m/z* 335 from BF cells. E. Observed fragmentation of Rha-C_10_ ion *m/z* 335 [M + H]^+^. F. Spectrum of ion *m/z* 320 from BF cells. G. Observed fragmentation of N-3-oxo-C_12_-HSL [M+Na]^+^. H. Spectrum of ion *m/z* 368 from BF cells. I. Observed fragmentation of C_4_-HSL [M+Au]^+^. Download FIG S3, TIF file, 1.3 MB. Copyright © 2022 Dodson et al. 2022 Dodson et al. https://creativecommons.org/licenses/by/4.0/ This content is distributed under the terms of the Creative Commons Attribution 4.0 International license.

10.1128/mbio.00191-22.9 TABLE S3 Nomenclature of discussed molecule abbreviations with structures. Download Table S3, DOCX file, 0.3 MB. Copyright © 2022 Dodson et al. 2022 Dodson et al. https://creativecommons.org/licenses/by/4.0/ This content is distributed under the terms of the Creative Commons Attribution 4.0 International license.

To consider whether MALDI analysis lysed cells, resulting in the ionization of intracellular molecules, whole-cell and cell lysate preparations were evaluated and compared (Fig. S6A). Ions visible only in the cell lysate suggested the ionization of intracellular molecules. Based upon apparent spectral differences between the two sample preparations (Fig. S6A), ions detected in this study were associated with the cell surface or extracellular matrix.

10.1128/mbio.00191-22.8 FIG S6 MALDI-TOF analysis in positive ion mode spectra. A. Stationary phase planktonic cells: intact whole cells (black) and cells lysed with acetonitrile (blue). Unique ions found to be in stationary-phase planktonic cells via MALDI-TOF were found in both whole cell and cell lysate samples. Yellow boxes show the ions only present in cell lysate but not in the intact whole cells. B. Cyclic-di-GMP spectrum of a commercially available cyclic-di-GMP standard. Cyclic-di-GMP was gold sputtered and analyzed in positive ion mode. Download FIG S6, TIF file, 1.2 MB. Copyright © 2022 Dodson et al. 2022 Dodson et al. https://creativecommons.org/licenses/by/4.0/ This content is distributed under the terms of the Creative Commons Attribution 4.0 International license.

Many unique ions observed in each subpopulation appeared to be either rhamnolipids or lactones. Ions unique to BF cells (Table 1) were judged as rhamnolipids: α-l-rhamnopyranosyl-β-hydroxyheptanoate (Rha-C_7_ *m/z* 293 [M+H]^+^), and α-l-rhamnopyranosyl-β-hydroxydecanoate (Rha-C_10_ *m/z* 335 [M+H]^+^). Herein, rhamnolipids will be denoted as Rha- followed by the aliphatic chain length, as described elsewhere (50) and in Table S3. The identity of Rha-C_7_ [M+H]^+^ was confirmed by comparing CID fragmentation of *m/z* 293 in BF cells to *m/z* 293 from a mono-rhamnolipid commercial standard (Fig. S3A to S3C). The BF unique ion, *m/z* 335 (Fig. S3D and S3E) fragmented similarly to *m/z* 293 (Rha-C_7_) (Fig. S3A to S3C), and comparably to published mass spectra of Rha-C_10_ [M+H]^+^ (49). The unique ECM cell ion, *m/z* 394, was identified as N-hexanoyl-l-homoserine lactone (C_6_-HSL [M-2H+Au]^+^), which was confirmed by comparing CID spectra to a spectrum from a commercial C_6_-HSL standard (Fig. S4A to S4C). Four SP cell unique ions were putatively identified as rhamnolipids (Table 1). These included mono-rhamnolipids: Rha-C_8_ (*m/z* 329 [M+Na]^+^), Rha-C_10_ (*m/z* 379 [M-H + 2Na]^+^), Rha-C_12:1_, (*m/z* 383 [M+Na]^+^), as well as a di-rhamnolipid, Rha-Rha-C_10-_C_12_ (*m/z* 702 [M+H+Na]^+^) (Fig. S5A to S5E, S5K to S5Q). CID fragmentation of mono- and di-rhamnolipid standards confirmed the identities of *m/z* 379 (Rha-C_10_) and *m/z* 702 (Rha-Rha-C_10-_C_12_) as similarities were seen between CID fragmentation of commercial standards and cell samples as well as previous publications (49, 51). Similarly, ions *m/z* 329 (Rha-C_8_), and *m/z* 383 (Rha-C_12:1_) were putatively identified based on comparable CID fragmentation patterns to those of confirmed rhamnolipids (Fig. S5A and S5B, S5K–S5L).

10.1128/mbio.00191-22.6 FIG S4 Positive ion mode MALDI CID of ECM unique ions. A. Spectrum of ion *m/z* 394 in ECM cells. B. Spectrum of ion *m/z* 394 from C_6_-HSL commercial standard from Cayman Chemical Company. C. Observed fragmentation of C_6_-HSL ion *m/z* 394 [M + 2H-Au]^+^. D. Spectrum of ion *m/z* 466 from ECM cells. E. Observed fragmentation of C_9:1_-2-alkyl-4-quinolone ion *m/z* 466 [M+Au]^+^. F. Spectrum of *m/z* 430 in ECM cells. G. Spectrum of *m/z* 482 in ECM cells. Download FIG S4, TIF file, 1.0 MB. Copyright © 2022 Dodson et al. 2022 Dodson et al. https://creativecommons.org/licenses/by/4.0/ This content is distributed under the terms of the Creative Commons Attribution 4.0 International license.

10.1128/mbio.00191-22.7 FIG S5 Positive ion mode MALDI CID of ions in SP cells. A. Spectrum of ion *m/z* 329 from SP cells. B. Observed fragmentation of Rha-C_8_ ion *m/z* 329 [M+Na]^+^. C. Spectrum of ion *m/z* 379 from SP cells. D. Spectrum of ion m/z 379 from R95 mono-rhamnolipid standard from AGAE Technologies. E. Observed fragmentation of Rha-C_10_ ion *m/z* 379 [M-H + 2Na]^+^. F. Spectrum of ion *m/z* 356 from SP cells. G. Observed fragmentation of C_12:1_-2-alkyl-4-quinolone ion *m/z* 356 [M-H + 2Na]^+^. H. Spectrum of ion *m/z* 260 from a 2-heptyl-3-hydroxy-4(1H)-quinolone (PQS) standard from Cayman Chemical Company. In the absence of a commercial standard for *m/z* 356 (C_12:1_-2-alkyl-4-quinolone [M-H + 2Na]^+^), a PQS standard was used to show expected quinolone fragmentation, which was observed on the alkyl chain. I. Spectrum of ion *m/z* 260 from SP cells. J. Observed fragmentation of PQS ion *m/z* 260 [M + H]^+^. K. Spectrum of ion *m/z* 383 from SP cells. L. Observed fragmentation of Rha-C_12:1_ ion *m/z* 383 [M+Na]^+^. M. Spectrum of ion *m/z* 702 (Rha-Rha-C_10_-C_12_) from SP cells. N. Spectrum of ion *m/z* 702 from R95 di-rhamnolipid standard from AGAE Technologies. Underlined ions represent fragments specific to Rha-Rha-C_12_-C_10_. Boxed ions represent fragments specific to Rha-Rha-C_10_-C_12_. O. Observed fragmentation of Rha-Rha-C_12_-C_10_/Rha-Rha-C_10_-C_12_ ion *m/z* 702 [M + H+Na]^+^. P. Observed fragmentation of Rha-Rha-C_12_-C_10_ ion *m/z* 702 [M + H+Na]^+^. Q. Observed fragmentation of Rha-Rha-C_10_-C_12_ ion *m/z* 702 [M + H+Na]^+^. R. Spectrum of ion *m/z* 404 in SP cells. Qualifier ions *m/z* 148 and *m/z* 163 are common fragments of rhamnolipids as shown in the inset. S. Spectrum of ion *m/z* 463 in SP cells. T. Spectrum of ion *m/z* 616 in SP cells. Download FIG S5, TIF file, 2.7 MB. Copyright © 2022 Dodson et al. 2022 Dodson et al. https://creativecommons.org/licenses/by/4.0/ This content is distributed under the terms of the Creative Commons Attribution 4.0 International license.

Additional unique ions were putatively identified as quinolones or rhamnolipids based on nominal mass and CID fragmentation patterns. Ion *m/z* 356 in SP cells was putatively identified as C_12:1_-2-alkyl-4-quinolone [M-H + 2Na]^+^ exhibiting fragmentation of the alkyl chain (Fig. S5F and S5G). To demonstrate the fragmentation patterns of quinolones, CID of ion *m/z* 260 [M+H]^+^ from a commercial standard, 2-heptyl-3-hydroxyquinolone (PQS) was performed and revealed comparable alkyl chain fragmentation (Fig. S5H and S5J). Similarly, ion *m/z* 466 in ECM cells was putatively identified as C_9:1_-2-alkyl-4-quinolone [M+Au]^+^ (Fig. S4D and S4E) with fragmentation of the alkyl chain resembling other work (49). CID fragment ions *m/z* 148 and *m/z* 163 were common among the identified rhamnolipids in this study. These fragments were present in CID data from ion *m/z* 404 in SP cells (Fig. S5R), suggesting that it is likely a rhamnolipid or a fragment of a rhamnolipid.

**FIG 6:**
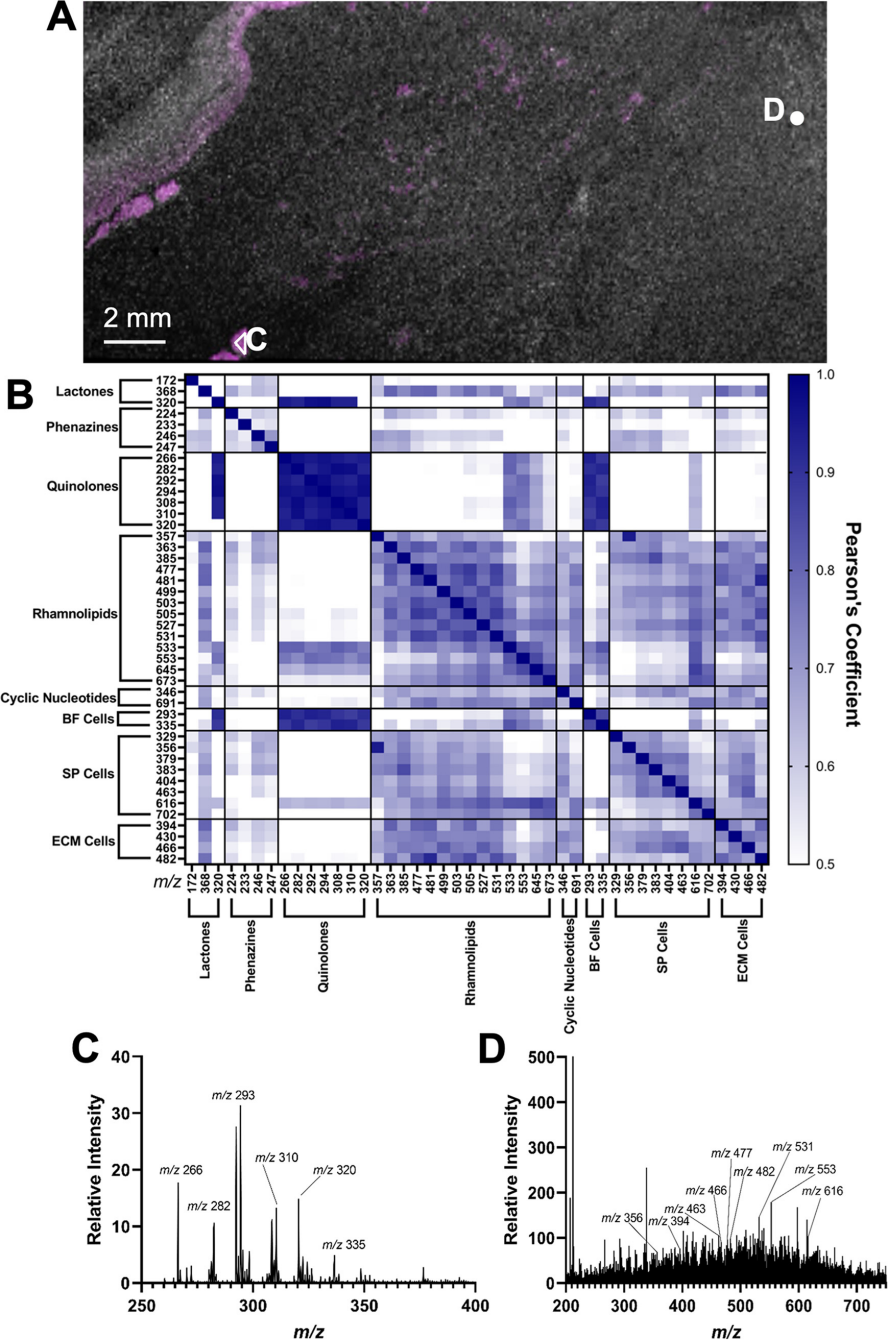
MALDI imaging of a representative PA14 biofilm grown on an ITO glass slide and gold sputtered. (A) MALDI imaging demonstrating localization of BF cell unique ions (magenta) and ECM/SP cell unique ions (white). MALDI-TOF spectra were extracted from two regions of interest, labeled C and D on the image. (B) Heat map showing Pearson’s coefficients of ions of significant co-localization via MALDI imaging. Pearson’s coefficients were calculated using the JACoP plug-in for Image J (54). (C) Spectrum extracted from region of interest, C, from MALDI imaging. Annotated ions on the spectrum are BF unique ions and selected quinolone ions with Pearson’s coefficients >0.7. (D) Spectrum extracted from region of interest, D, from MALDI imaging. Annotated ions on the spectrum are ECM and SP unique ions and selected rhamnolipid ions with Pearson’s coefficients >0.7.

Via MALDI imaging of an adherent biofilm, cell subpopulation localizations were assessed. A static PA14 biofilm was grown as described in the Materials and Methods section, including a submerged indium-titanium oxide (ITO) coated glass slide that permitted biofilm adherence to one surface. After biofilm growth, the ITO slide was removed, allowing biomass collection onto the slide’s surface. The biofilm-coated ITO slide was subsequently dried, gold sputtered, and imaged via MALDI-TOF. To demonstrate localization of cell subpopulations, MALDI images were generated and the unique ion images (Table 1) were overlaid (Fig. 6A).

MALDI imaging of ions representing known P. aeruginosa signaling molecules (phenazines, rhamnolipids, lactones, quinolones, and cyclic nucleotides) (22, 49, 52, 53) were generated to determine their localization within the biofilm (Fig. 6B). Many of these ions localized with specific cell subpopulations (Fig. 6C and D and Table S4). Co-localization of unique ions and quorum sensing molecule ions were calculated with Pearson’s coefficients using JACoP, an Image J plug-in (54) (Fig. 6B and Table S4). Spectra from regions of interest (Fig. 6C and D) via MALDI imaging (Fig. 6A) demonstrate that BF unique ions (*m/z* 293 and 335) co-localize with expected quinolone ions, *m/z* 266, 282, 292, 294, 308, 310, and 320 with Pearson’s correlation coefficients above 0.9 (Fig. 6B and C and Table S4). Cyclic-di-GMP ion *m/z* 691 [M+H]^+^ (Fig. S6B) (55), localized with ions, *m/z* 616, 702, 430, and 482, unique to ECM or SP cells. In addition to the rhamnolipid ions identified as unique ions from SP or BF cells, other mono- and di-rhamnolipid ions were detected as [M+H]^+^ or [M+Na]^+^ (Fig. 6B and D). The ion, *m/z* 320, predicted to be N-3-oxo-C_12_-HSL [M+Na]^+^ (Fig. S3F and S3G), co-localized with ions unique to BF cells (Pearson’s correlation coefficients; *m/z* 293: 0.911, *m/z* 335: 0.888). The examination of small-molecule localizations with cell subpopulations can give insight into signaling patterns in biofilms.

10.1128/mbio.00191-22.10 TABLE S4 Calculated Pearson’s coefficients for all ions of interest. Blue boxes indicate Pearson’s coefficient value >0.7 and green boxes indicate Pearson’s coefficient value >0.9. Black represents an ion being compared against itself. Download Table S4, DOCX file, 0.1 MB. Copyright © 2022 Dodson et al. 2022 Dodson et al. https://creativecommons.org/licenses/by/4.0/ This content is distributed under the terms of the Creative Commons Attribution 4.0 International license.

## DISCUSSION

Differences in cell morphologies of single-species bacterial cultures historically have been overlooked or underappreciated. This is partly due to a lack of tools to separate and elucidate these cellular phenotypes. The biofilm harvesting method described here details the isolation of three phenotypically distinct biofilm cell subpopulations for subsequent analysis. Previous studies have reported only two subpopulations of cells (5, 56). Utilizing large glass petri dishes promoted complex biofilm structures growing at both solid-liquid and liquid-air interfaces to form in quantities sufficient for isolation of lower-abundant cell subpopulations, contrasting to common smaller culture volumes (36, 57). The cell separation method is adaptable to efficiently and inexpensively isolate distinct cell subpopulations of sessile biofilms in sufficient quantities to probe their morphologies and phenotypes and could permit separation of cells with less cytosolic density (58).

In P. aeruginosa, the switch from planktonic to biofilm lifestyles is regulated by genes associated with biofilm phenotypes (15, 59). For example, the gene *algR*, part of the *algR/algZ* two-component system responsible for alginate production, controls P. aeruginosa biomass production by indirectly modulating cyclic-di-GMP (59). These results corroborate our findings as *algR* transcripts are higher (Fig. 4A) in cells which exhibit higher biomass forming abilities (Fig. 3C) and pro-biofilm traits: wrinkled colony morphology (60) (Fig. 3E), lower swarming (37) (Fig. 3A and B), and exopolysaccharide production (35, 61) (Fig. 3D). Since SP and ECM cell unique ions co-localize with ion *m/z* 691 (Fig. 6B) (55), SP cells exhibit higher *algR* transcript levels, and SP and ECM cells most correlate with pro-biofilm behaviors compared to other subpopulations, it is possible that SP and ECM cells have higher cyclic-di-GMP levels than other cell subpopulations.

Previous literature indicates that cells in planktonic phase and biofilms have differing antibiotic susceptibility, with planktonic P. aeruginosa cells being more sensitive to antibiotic treatment (27). Hypotheses state that cells in biofilms are more tolerant to antibiotics due to their inability to penetrate the biomass (62). This study confirms these findings, as higher concentrations of colistin or tobramycin were needed to dissociate biofilms from a surface (Fig. 5C) than were required to prevent their accumulation (Fig. 5A and Table S2). However, the data suggest that both biomass penetration and inherent cellular antibiotic tolerance affects the response of cells in a biofilm to antibiotic treatment, as generally, cell subpopulations were differentially affected by antibiotics (Fig. 5). The response of cell subpopulations to antibiotic challenge (Fig. 5) do not correlate directly with biomass production (Fig. 3C). Although SP cells accumulated more biomass (Fig. 3C and Table S1), established SP biomass was generally more susceptible to higher concentrations of antibiotic treatment in comparison to the BF subpopulation (Fig. 5C and D and Table S2) which produced overall less biomass (Fig. 3C). Thus, the composition or structure of biofilms derived from each cell subpopulation differs. This finding is further confirmed via the different extracellular matrices observed by SEM (Fig. 1A) and MALDI imaging (Fig. 6). More effective biomass dispersal strategies may be developed upon understanding the contributions and mechanisms involved in biomass production and structure.

P. aeruginosa mature biofilm extracellular matrices consist of biomolecules such as rhamnolipids (9). This work reports Rha-C_7_ (*m/z* 293 [M+H]^+^) for the first time in P. aeruginosa as rhamnolipid alkyl chains predominately are C_8_ to C_12_ in length (63); however, two P. aeruginosa isolates, have been reported to possess di-rhamnolipids with C_6_ alkyl chains (51, 64). Thus, rhamnolipid chain lengths shorter than C_8_ exist. Here, Rha-C_7_ was detected by MALDI-TOF. The high energy gold matrix, also used as an internal standard (49), aided the ionization of molecules in positive ion mode. However, post-source decay or high laser-induced fragmentation are also potential causes of rhamnolipid alkyl chain breakage which could result in the detection of rhamnolipid ions with shorter alkyl chains than the endogenous parent rhamnolipid. In this study, rhamnolipids fragment at the glycosidic bond, the carboxylate, or result in the loss of water (Fig. S3A to S3E, S5A to S5E, S5K to S5R). This reasonably suggests that Rha-C_7_ could be endogenous to P. aeruginosa PA14 biofilms, though this ion (*m/z* 293 [M+H]^+^) could also be a fragment of larger mono- or di-rhamnolipids.

Quorum sensing molecules affect the expression of potentially charged surface molecules and resulting cell physiology (14). This suggests that identifying molecules unique to cell subpopulations could lead to understanding the relationship between molecular signals and specific cell phenotypes, biofilm locations, and roles. C_4_-HSL production depends on RhlI activity (65), which correlates with the observation of significantly higher *rhlI* transcript levels in SP cells compared to planktonic cells (Fig. 4A). Additionally, co-localization (Table S4) of C_4_-HSL (*m/z* 368) (Fig. S3H and S3I) and C_6_-HSL (*m/z* 394) (Fig. S4A to S4C) with ions unique to high biofilm forming ECM and SP cells (*m/z* 379, 383, 404, 430, 463, 466, 482) corroborates previous work correlating extracellular C_4_-HSL and C_6_-HSL levels with biofilm formation (66). N-3-oxo-C_12_-HSL levels were previously demonstrated to be constant during biofilm development and were thought to aid in biofilm initiation (66). Consequently, the co-localization of expected N-3-oxo-C_12_-HSL ion (*m/z* 320) (Fig. S3F and S3G) with ions unique to BF cells (Fig. 6B) indicates that BF cells likely play a key role in the initiation of biofilms.

In bacteria, the mechanisms promoting phenotypic heterogeneity have been understudied in comparison to differentiation in multicellular organisms. However, these studies are essential to facilitate the understanding of microbes and may allow for the discovery of new bacterial treatments, especially those for preventing the formation of biofilms or initiating biofilm dispersal. Our work describes a platform to enable the identification, isolation, and characterization of different cell subpopulations in biofilm-producing bacteria. Subpopulations of bacterial cells exhibit morphologic and phenotypic variations, such as unique surface molecules, suggesting these cells play different roles in the biofilm community. MALDI imaging analysis of ionizable cell surface molecules permitted the mapping of distinct localized cell subpopulations within a biofilm. These unique surface molecules could potentially be targeted with novel cellular probes to further study specific cell phenotypes. Small signaling molecules co-localize with unique ions from cellular subpopulations suggesting possible regulatory roles and cell specificities previously undescribed. Understanding the individual roles of cell subpopulations is crucial to unraveling the complicated mechanisms that are involved in the initiation and formation of biofilms. The knowledge of cellular responses establishing biofilm cell subpopulations can aid in dispersing biofilms involved in infections and environmental contamination. Previous works have described some biochemical pathways involved in biofilm production and dispersal, but the elucidation of their interconnection has been difficult, largely due to the complexity of the biofilm matrix. However, we were able to isolate and study distinct cell subpopulations in heterogeneous bacterial environments, and future similar studies can be used to characterize and unravel the complex pathways associated with bacterial life cycles.

## MATERIALS AND METHODS

### Cell cultures, culture conditions, and reagents.

Pseudomonas aeruginosa UCBPP-PA14 wild-type cells were gifted from the laboratory of Peter C. Dedon at MIT were propagated and maintained in Luria-Bertani (LB) broth, Miller (Fisher BioReagents, NJ, USA) and LB agar, Miller, granulated (Fisher BioReagents, NJ, USA) dissolved in Milli-Q water (Millipore, MA, USA).

### Cell growth and harvesting.

A single colony propagated in LB broth and grown at 37°C with orbital shaking was classified as one biological replicate. Stationary-phase planktonic cells were grown in LB broth overnight (OD_600_ ≥1.5). Mid-log cells were grown by re-inoculation of stationary-phase cells in LB broth and grown as stated above until OD_600_ reached 0.3. Cells were harvested by centrifugation at 11,000 × *g* at 4°C for 15 min. The Multiskan Spectrum microplate and cuvette reader with SkanIt RE for MSS 2.4.4 software (Thermo Electron Corporation, MA, USA) was used for turbidity measurements.

For biofilm growth, cells were propagated as described above to stationary-phase and diluted to OD_600_ 0.3 ± 0.01 and further diluted 1:500 into 50 mL of sterile LB broth and grown without shaking at 37°C for 48 h in Pyrex 150 × 20 mm petri dishes.

Attached biofilms were removed and 1/10th volume of sterile 0.9% saline was added and briefly vortexed. The biofilms were centrifuged at 11,000 × *g* at 4°C for 45 min. Supernatant was removed for further centrifugation. The centrifuged pellet consisted of two separable layers: the top layer comprising extracellular matrix, including ECM cells, and the bottom layer comprising BF cells. The two layers were separated by gentle shaking. The BF cell pellet was washed with sterile saline and centrifuged as before. Figure 1B was created with BioRender.com. To calculate cell frequency in the biofilms, respective cell pellet weights were measured. The cell frequency is reported as cell weight (g) to biofilm volume (mL) and reported as an average of 3 representative biological replicates.

The extracellular matrix was added to sterile saline containing 750 units DNase I from bovine pancreas (Part number D5025-150KU) (Sigma-Aldrich, MO, USA), 25 units alginate lyase (Part number A1603, powder, ≥10,000 units/g) (Sigma-Aldrich Corp., MO, USA) and 1 mM MgCl_2_ (99% ACS reagent, Acros Organics, NJ, USA). The enzymatic digest was incubated with orbital shaking at 37°C for 5 h. The ECM cells were then centrifuged at 10,000 × *g* at 4°C for 30 min. The ECM cells were washed twice as mentioned above.

The supernatant was centrifuged at 28,000 × *g* at 4°C overnight, unless otherwise noted. The resulting pellet was solubilized and washed in sterile saline and pelleted by centrifugation at 18,000 × *g* at 4°C for 30 min. All resulting experiments were done with freshly harvested planktonic, BF, ECM, and SP cells, unless otherwise noted. The cultures were standardized to an OD_600_ of 0.3 ± 0.01 in either sterile saline or LB broth.

### LB agar plating and light microscopy.

Cells were plated on LB agar. Plates were incubated at 37°C overnight. Light microscopy was performed on Olympus SZX7 Stereoscopic Light Microscope with an Olympus SC100 camera attachment (Olympus Scientific Solutions Americas Corp., USA). There were *n* ≥ 9 biological replicates per subpopulation.

### Swarming assays.

Swarming assay procedures were adapted from previous work (67). M9 minimal media 0.4% agar plates were poured in a sterile environment, UV-irradiated for 20 min, and air dried for 40 min. Promptly, 5 μL of cultures standardized to OD_600_ 0.3 were pipetted on the middle of the plates and incubated at 37°C for 24 h. Pictures of the plates were taken by an Azure C500 equipped with an 8.3MP Camera (Azure Biosystems, Inc., CA, USA) and the swarming areas were quantified using ImageJ1. There were *n* ≥ 15 biological replicates per subpopulation.

### Zeta potential and DLS particle size analysis.

The cell zeta potential was measured in 0.02 μm filtered saline with gold electrode Omega cuvettes (Anton Paar, Graz, Austria). For DLS measurements, a 1:50 dilution of the standardized cultures in filtered saline was read in polystyrene cuvettes. The zeta potential and DLS were measured using Anton Paar Litesizer 500 and Kalliope software (Anton Paar, Graz, Austria). Zeta potential measurements used standard aqueous method settings. For particle size analysis, samples were equilibrated at room temperature in aqueous 154 mM NaCl (refractive index 1.3323), and material refractive index set at 1.388 for bacteria. For DLS and zeta potential experiments, *n* ≥ 8 biological replicates per subpopulation.

### RT-qPCR.

Total nucleic acid was isolated from each cell subpopulation (68). To obtain template for qPCR standard curves, PCR was performed with qPCR primers then the PCR products were run on a 0.8% agarose gel. The pure PCR product was isolated from the gel using the Silica Bead DNA Gel Extraction kit (ThermoFisher) according to the manufacturer’s instructions. Gel-isolated PCR products were diluted between 1.0 × 10^8^ and 1.0 × 10^1^ copy per reaction for qPCR analysis.

For RT-qPCR sample preparation, total nucleic acid was isolated as above and treated with DNase, RNase-free (Roche) according to the manufacturer’s instructions to obtain pure total RNA. The LunaScript RT SuperMix kit (New England Biolabs) was used to create cDNA libraries from biological triplicates of all cell subpopulations. The expression of the target genes was analyzed with Luna Universal qPCR Master Mix (New England Biolabs) on a Bio-Rad iCycler iQ. The expression of *lasI*, *lasR*, *rhlI*, *rhlR*, *algR*, and *algZ* was analyzed utilizing *fabD* as a reference gene (43, 44). The primer sequences used for standard curve PCR and RT-qPCR of *lasI*, *lasR*, *rhlI* (69), *rhlR* (70), *algR* and *algZ* (42) transcripts were previously reported and *fabD* primer sequences used for standard curve PCR and RT-qPCR were as follows:

Forward: 5′- GGTCCAGAATGGACCTGAAGAG -3′

Reverse: 5′- CGATCGAAACCGTGAGGATGGC -3′.

There were *n* ≥ 3 biological replicates per subpopulation.

### Crystal violet biofilm assays.

The cultures were standardized to an OD_600_ of 0.3 and inoculated 1:100 in LB broth and the crystal violet assay was performed as previously described (36). Cultures in 96-well plates were statically grown at 37°C for 24 h. There was *n* ≥ 12 biological replicates, with 4 technical replicates per biological replicate, per subpopulation (See Table S1). For biofilm inhibition assays, tobramycin (Part number T4014) (Sigma-Aldrich, MO, USA) or colistin sulfate salt (Acros Organics, NJ, USA) were added at various concentrations. In LB broth, the MIC of tobramycin for PA14 is 1 μg/mL (2.1 μM) (71) and 0.5 μg/mL for colistin (72). For biofilm dissociation assays, after the initial 24 h of growth, the LB broth was replaced with new LB broth containing either tobramycin or colistin at various concentrations and allowed to grow for an additional 24 h at 37°C. Resulting biofilms were quantified as previously described (36). There were *n* ≥ 4 biological replicates, with 4 technical replicates per biological replicate, per subpopulation (See Table S2). Two-way ANOVA with *post hoc* Tukey’s tests for multiple comparisons were performed to determine significance.

### Congo red assays.

The cultures were standardized to an OD_600_ of 0.3 and inoculated 1:1000 in LB broth, Congo red was added to a final concentration of 40 μg/mL (35) and grown overnight at 37°C with shaking. After growth, cultures were centrifuged to remove cells and the supernatant was measured at A_490_ in a cuvette. There were *n* ≥ 4 biological replicates per subpopulation.

### Growth curves.

The cultures were standardized and further diluted 1:1000 and grown in LB broth with orbital shaking. OD_600_ readings occurred every 20 min for 12 h at 37°C. There were *n* ≥ 4 biological replicates with 4 technical replicates per biological replicate per subpopulation.

### SEM imaging of cells and biofilms.

Cells were fixed overnight at 4°C in 2.5% glutaraldehyde buffered with 0.1 M sodium cacodylate, pH 7.4 (Electron Microscopy Sciences, PA, USA). Cells were washed with a gradient of phosphate-buffered saline (10× and 5× in Milli-Q water) (Part number BP399) (Fisher BioReagents, NJ, USA) then DI water prior to an ethanol dehydration gradient in DI water. Subsequently, a gradient using hexamethyldisilazane (HMDS) (SPI Supplies, PA, USA) in ethanol was used for chemical dehydration (73). The samples were coated with 5 to 7 nm of gold and imaged with the JEOL JSM-7500F Cold Cathode Field Emission Microscope (JEOL Ltd., Tokyo, Japan).

Biofilms to be imaged via SEM were inoculated as above in 6-well plates with the addition of a plastic coverslip on the bottom of the wells. After 24 h of growth, the wells were allowed to air dry in a sterile environment. The biofilm was fixed with 2% paraformaldehyde (Fisher Chemical, NJ, USA) overnight. The coverslip with biofilm was subjected to the dehydration gradient, gold sputtered, and analyzed as described above.

### Brightfield and fluorescence microscopy.

Each cell subpopulation was harvested, then washed with 0.9% NaCl. Cells were heat fixed onto a microscope slide, then stained with Syto RNASelect Green fluorescent cell stain (Invitrogen) and propidium iodide (Acros Organics) for live/dead staining. Cells were imaged on a Nikon Eclipse Ti-S inverted microscope using a 100× objective.

### MALDI MS analysis of cells and biofilms.

MALDI experiments were performed using a Bruker UltrafleXtreme MALDI-TOF/TOF with flexControl software. Experiments were conducted in reflectron positive mode at a laser intensity of 40%, 50%, or 70% and an *m/z* range 0-2000. Random walk was set to partial sample and a sum of at least 10,000 laser pulses were taken per sample unless noted otherwise. Mass lists were generated (S/N ≥ 3) using flexAnalysis Bruker software.

All cells were isolated as described above then plated on an ITO slide for MALDI Imaging and subsequently air-dried. To determine if ions detected were from cell lysate or whole cells, cell lysate was prepared by suspending cells in acetonitrile, while whole cells remained intact and were resuspended in sterile saline for analysis. The slide was gold sputtered for 3 s (1 to 2 nm) and the instrument was calibrated as previously described (49). Gold and LB broth associated ions were manually identified and subtracted from the final cell spectra. For CID, the cells were prepared and gold sputtered as described above and argon was used as collision gas with a pressure of 5 × 10^−6^ mbar. Detection of unique ions were in half of the biological replicates tested (*n* ≥ 8) from at least three different days.

A 50 mL biofilm was inoculated and grown as described above with the addition of an ITO MALDI slide submerged at the bottom of the inoculum. After growth the glass plate was carefully removed from the bottom of the biofilm and allowed to air dry. Reference marks were made on the plate to guide the automated AutoXecute Bruker imaging system. The laser diameter was set to 50 μm and raster width at 75 μm. Fuzzy control, MS/Parent Mode was set to “On” with 50 satisfactory shots per raster spot. FlexImaging Bruker software was utilized to analyze spectra and heat maps, which were generated using root means squared (RMS) normalization. MALDI imaging co-localization statistics (Pearson’s coefficients) were calculated using the JACoP Image J plug-in (54).

Mono-rhamnolipid (R95) and di-rhamnolipid (R95) standards were purchased from AGAE Technologies, Oregon. N-hexanoyl-l-Homoserine lactone (C_6_-HSL) and 2-heptyl-3-hydroxy-4(1H)-quinolone (PQS) standards were purchased from Cayman Chemical Company, Michigan. Cyclic-di-GMP standard was purchased from Sigma-Aldrich, Missouri. All standards were suspended in sterile saline, spotted on an ITO slide, then gold sputtered for MALDI-TOF or MALDI CID analysis.

### Statistics.

Welch’s unpaired two-tailed *t* tests were performed for most analyses (without statistical corrections multiple comparisons). Two-way ANOVA with *post hoc* Tukey’s tests for multiple comparisons were performed for antibiotic assays. When applicable, Grubb’s tests were performed to remove outliers. *P* values ≤ 0.05 were deemed significant and *P* values ≥ 0.05 were deemed nonsignificant (ns). Nonsignificant *P* values were either represented by (ns), numerical *P* values, or were not indicated on graphs. All *P* values, t-values, degrees of freedom (df), technical replicates, and biological replicates (n) from statistical analyses were reported in the respective figure captions. Pearson’s coefficients were obtained for MALDI imaging heat maps to determine significant levels of co-localization between ions of interest (54).
