# Supplementary material for: Characterization of Distinct Biofilm Cell Subpopulations and Implications in Quorum Sensing and Antibiotic Resistance
Source: mBio. 2022 Jun 13;13(3):e00191-22. doi: 10.1128/mbio.00191-22 (PMC9239111; doi:10.1128/mbio.00191-22)
Supplement: TABLE S1 [file mbio.00191-22-s0001.docx]

| **Table S1: Comparison of the zeta potential, biomass accumulation, CFU/mL, and cell frequencies between subpopulations** | | | | | | | | | | | |
| --- | --- | --- | --- | --- | --- | --- | --- | --- | --- | --- | --- |
| Cell subpopulations | Zeta Potential (^−^mV) | | | % Biomass normalized to stat. planktonic cells | | | CFU/mL (in 10^8^) | | | | Cell frequency in whole biofilm (g/mL) |
|  | Average ± Standard deviation | Biological replicates | P values | Average ± Standard deviation | Biological replicates | P values | Average ± Standard deviation | Biological replicates | P values | Frequency % |  |
| Stationary planktonic cells | 17.22 ± 0.84 | 21 | NA | 100 ± 0 | 12 | NA | 1.84 ± 1.24 | 9 | NA | 100 |  |
| Stationary planktonic RSCV cells |  |  |  |  |  |  | 0 | 9 |  | 0 |  |
| Mid-log cells | 17.74 ± 0.95 | 8 | 0.1643 |  |  |  |  |  |  |  |  |
| BF cells | 18.10 ± 0.631 | 11 | 0.0051 | 171.9 ± 28.3 | 12 | <0.001 | 1.08 ± 0.361 | 11 | 0.1087 | 93.18 | 7.7 x 10^-3^ ± 3.9 x 10^-3^ |
| BF RSCV cells |  |  |  |  |  |  | 0.0736 ± 0.041 | 11 |  | 6.82 |  |
| ECM cells | 15.70 ± 0.48 | 11 | <0.001 | 215.8 ± 30.0 | 17 | <0.001 | 1.82 ± 1.57 | 12 | 0.9743 | 89.86 | 25.7 x 10^-3^ ± 0.90 x 10^-3^ |
| ECM RSCV cells |  |  |  |  |  |  | 0.182 ± 0.16 | 12 |  | 10.14 |  |
| SP overnight | 23.41 ± 2.93 | 12 | <0.001 | 360.8 ± 80.9 | 12 | <0.001 | 0.28 ± 0.202 | 10 | 0.0053 | 79.64 | < 1.0 x 10^-3^ |
| SP RSCV cells |  |  |  |  |  |  | 0.057 ± 0.059 | 10 |  | 20.36 |  |
| SP 3 hour | 22.27 ± 0.0012 | 3 | <0.001 |  |  |  | 1.91 ± 1.1 | 9 | 0.931 |  |  |
| SP 6 hour | 19.94 ± 0.001 | 2 | <0.001 |  |  |  |  |  |  |  |  |
| All P values calculated from unpaired two-tailed t tests comparing cell subpopulations to stationary planktonic cells, when applicable. SP cells were collected for analysis after centrifugation for either; 3 hours, 6 hours, or overnight (13-16 hours) at 28,000 x g. Zeta potential data represented as a mean of biological replicates ± standard deviation. % Biomass accumulation was stained with crystal violet and absorbance taken at 550 nm. Absorbance readings were normalized to absorbance readings of stationary planktonic cell biomass accumulation. % Biomass accumulation data is the average of biological replicates with 4 technical replicates per biological replicate. CFU/mL are an average of biological replicates plated on LB agar. Error is represented as the standard deviation in CFU/mL. Frequency % values represent the average percentage of normal colonies versus their respective RSCV colonies (e.g. frequency % of BF cells (93.18%) and frequency % of BF RSCV cells (6.82%) equate to 100%). Cell frequency in whole biofilm is calculated as an average and standard deviation of 3 representative biological replicates of the dry weight of cells per total volume of biofilm. | | | | | | | | | | | |
