# Supplementary figures and images for: Characterization of Distinct Biofilm Cell Subpopulations and Implications in Quorum Sensing and Antibiotic Resistance
Source: mBio. 2022 Jun 13;13(3):e00191-22. doi: 10.1128/mbio.00191-22 (PMC9239111; doi:10.1128/mbio.00191-22)

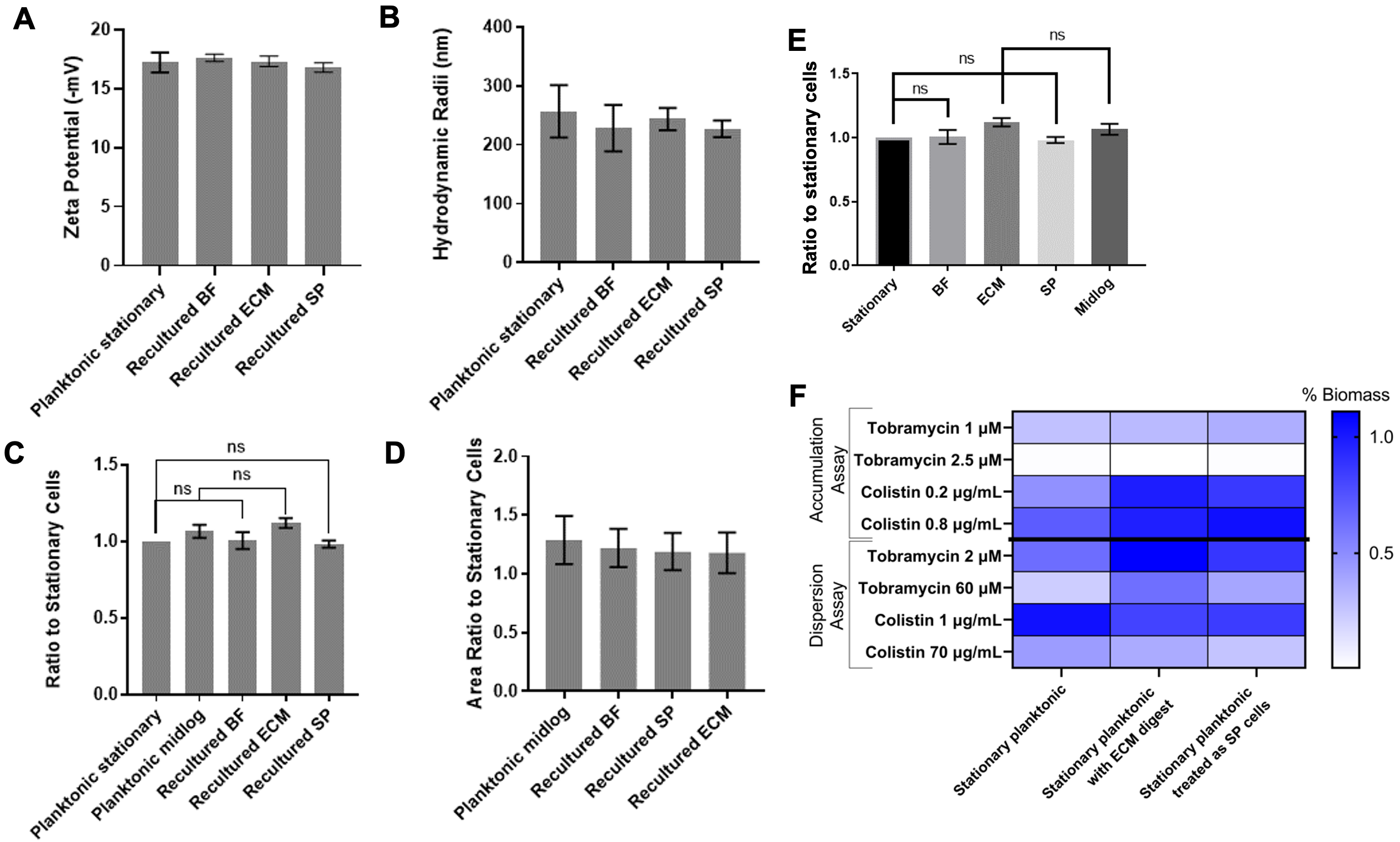

Supplement: FIG S2 [file mbio.00191-22-s0010.tif]

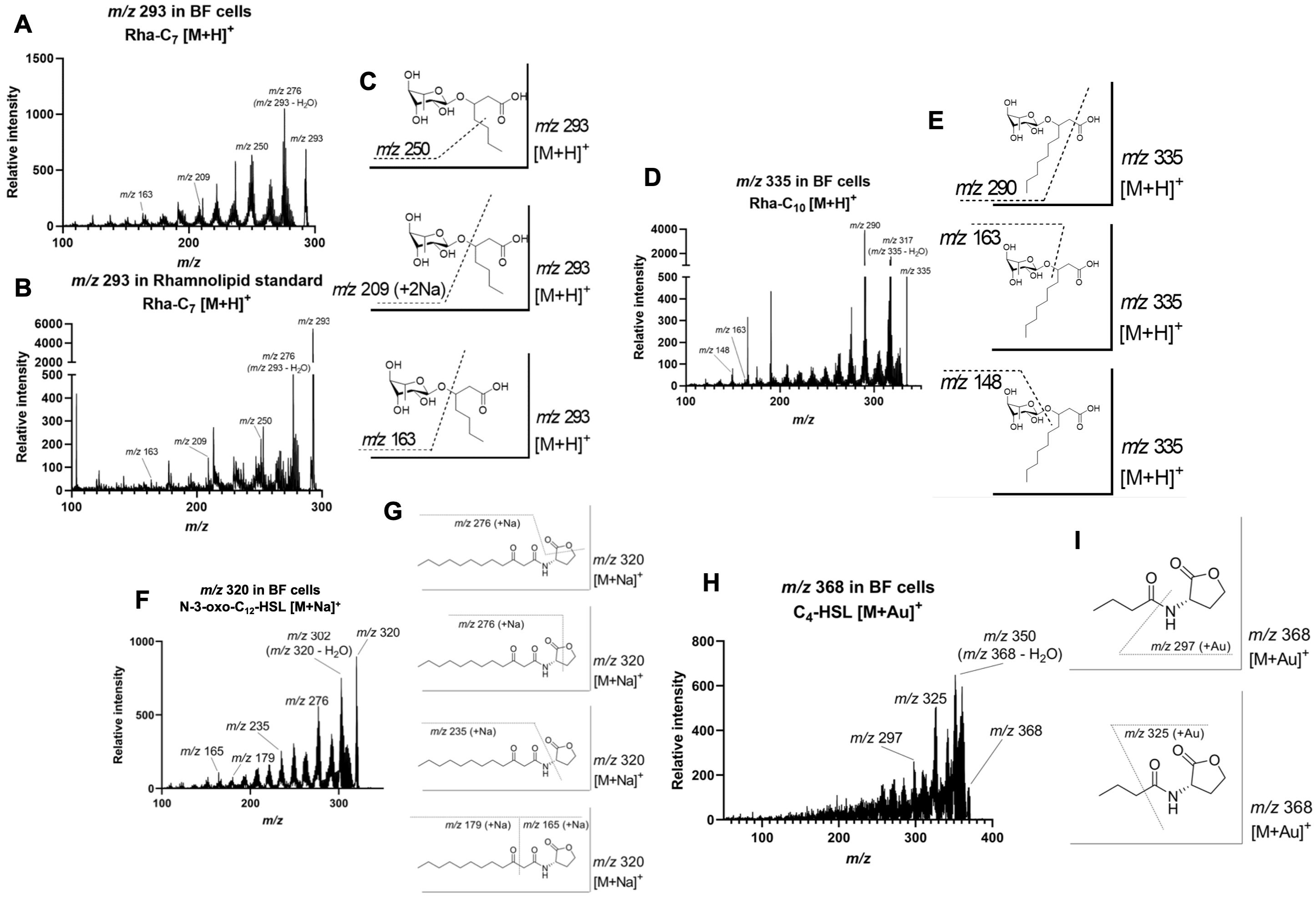

Supplement: FIG S3 [file mbio.00191-22-s0005.tif]

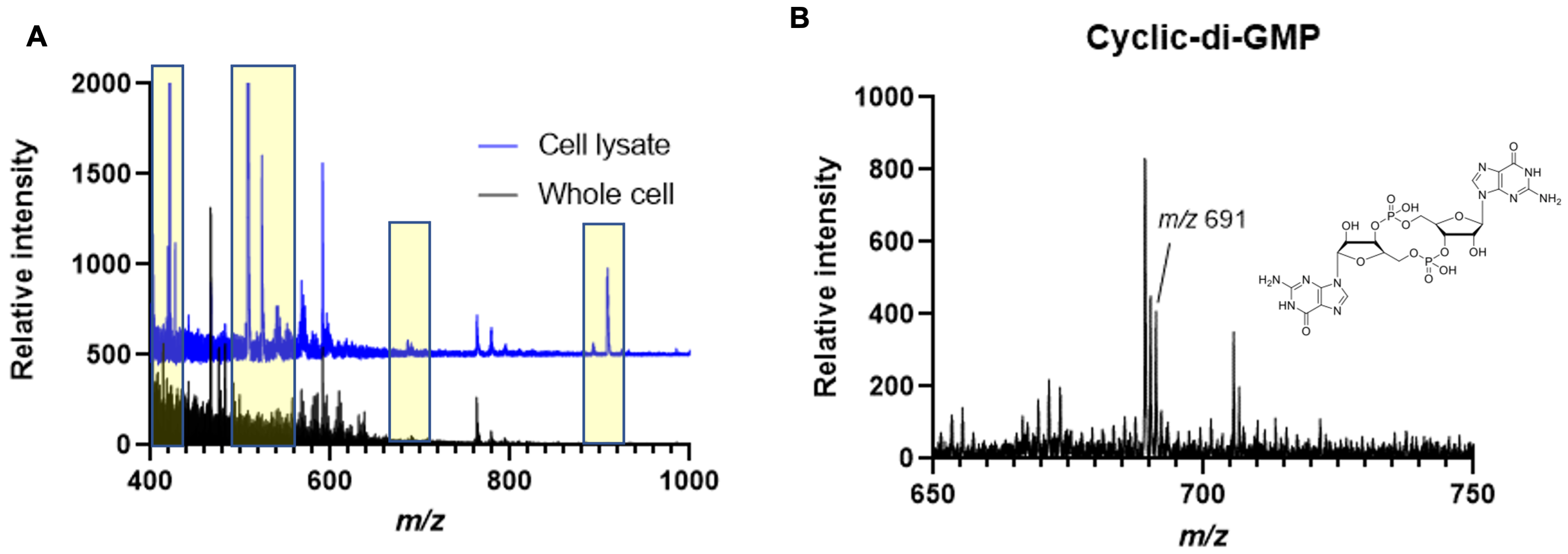

Supplement: FIG S6 [file mbio.00191-22-s0008.tif]

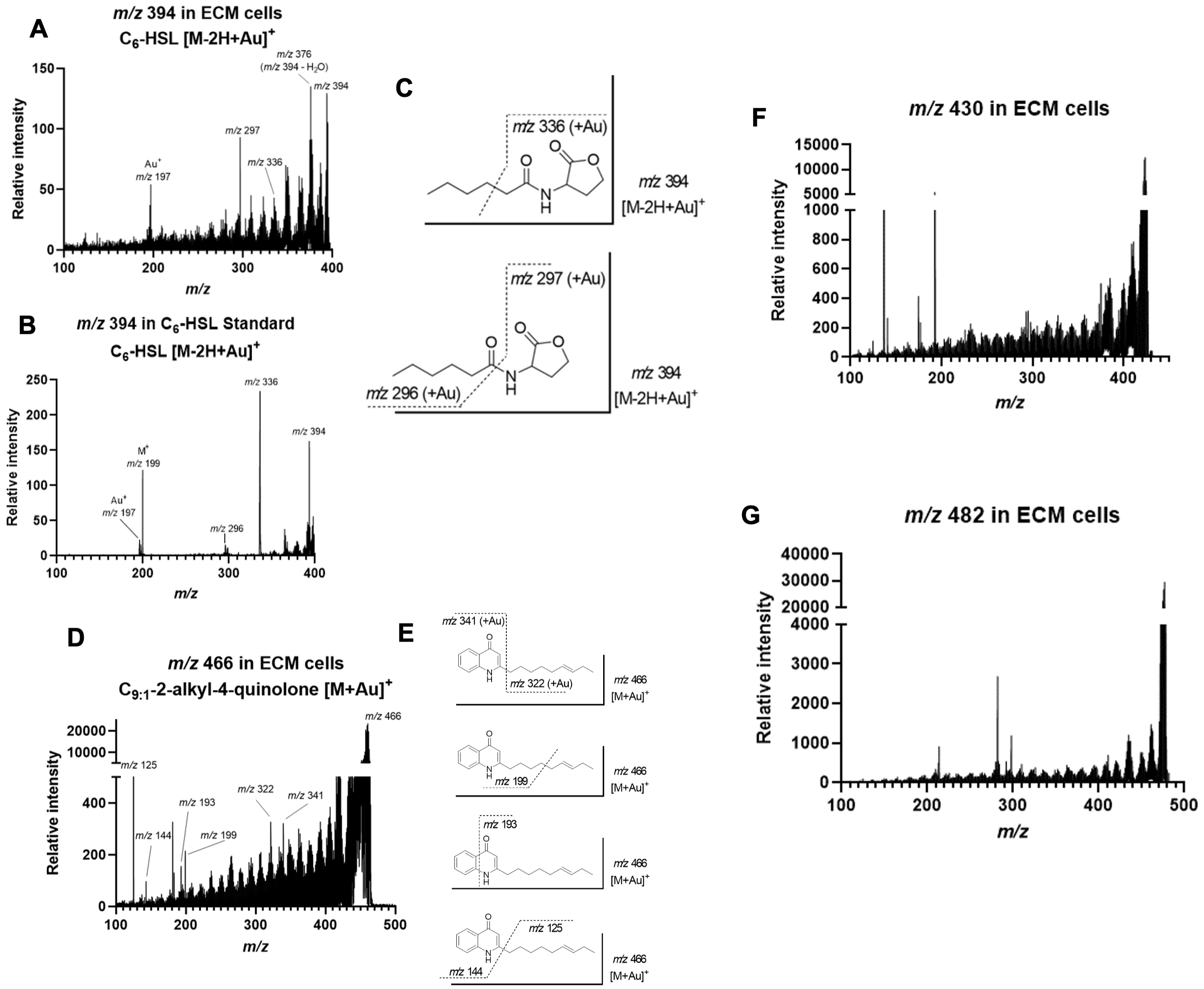

Supplement: FIG S4 [file mbio.00191-22-s0006.tif]

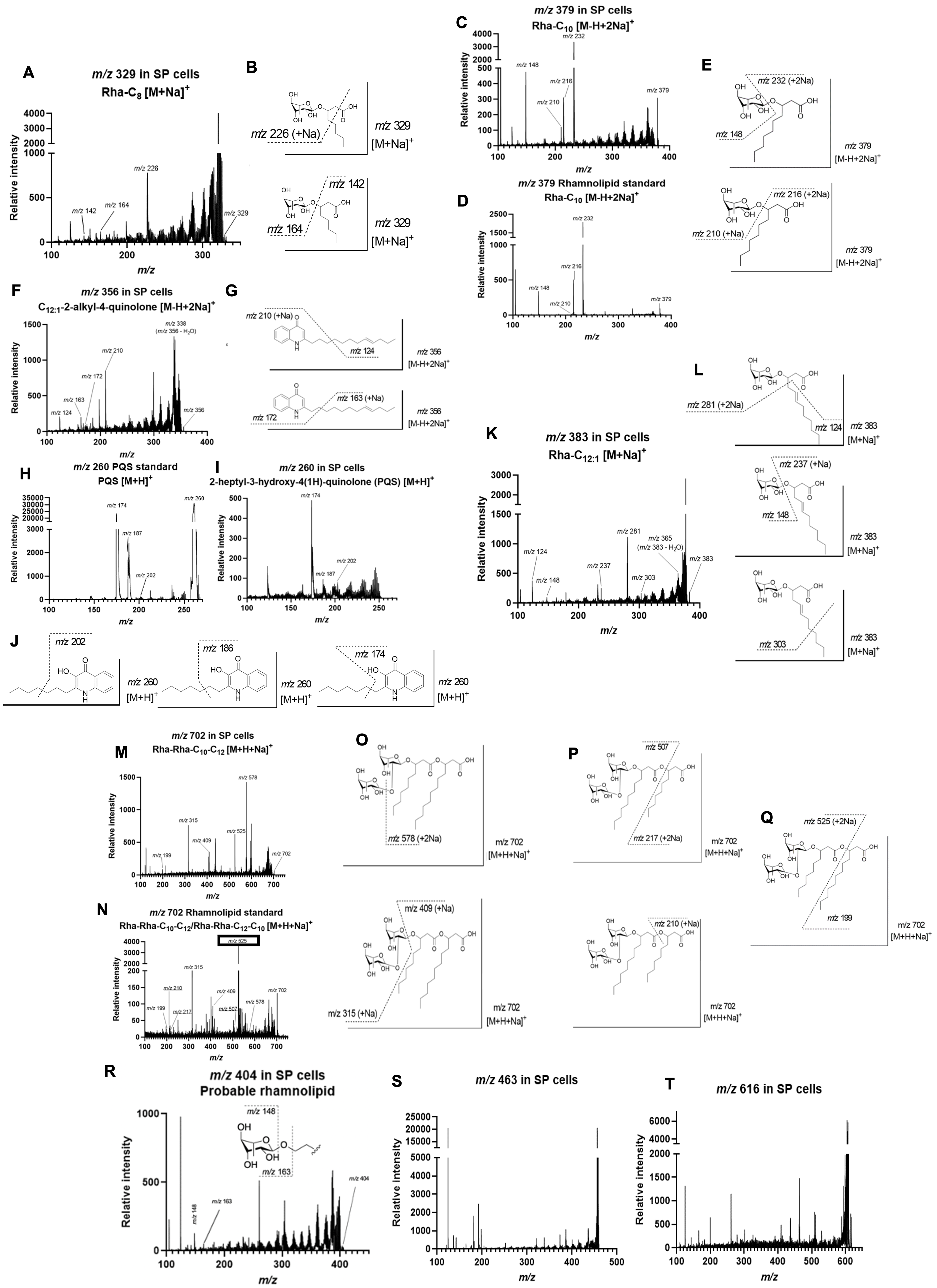

Supplement: FIG S5 [file mbio.00191-22-s0007.tif]
