# Supplementary material for: Characterization of Distinct Biofilm Cell Subpopulations and Implications in Quorum Sensing and Antibiotic Resistance
Source: mBio. 2022 Jun 13;13(3):e00191-22. doi: 10.1128/mbio.00191-22 (PMC9239111; doi:10.1128/mbio.00191-22)
Supplement: TABLE S2 [file mbio.00191-22-s0002.docx]

| **Table S2: Crystal Violet Antibiotic Assays** | | | | | | | | | | | | | | | | | | | | | | | | | | | | | | | | | | | |
| --- | --- | --- | --- | --- | --- | --- | --- | --- | --- | --- | --- | --- | --- | --- | --- | --- | --- | --- | --- | --- | --- | --- | --- | --- | --- | --- | --- | --- | --- | --- | --- | --- | --- | --- | --- |
| **Tobramycin Accumulation Assay** | | | | | | | | | | | | | | | | | | **Colistin Accumulation Assay** | | | | | | | | | | | | | | | | | |
|  | **1.0 μM** | | | **1.5 μM** | | | **2.0 μM** | | | **2.5 μM** | | | | **3.0 μM** | | | | **0.2 μg/mL** | | | | **0.4 μg/mL** | | | | **0.6 μg/mL** | | | **0.8 μg/mL** | | | | | **1.0 μg/mL** | |
| Samples | % Biofilm Accumulation | n | | % Biofilm Accumulation | | n | % Biofilm Accumulation | | n | % Biofilm Accumulation | | | n | % Biofilm Accumulation | | | n | % Biofilm Accumulation | | | n | % Biofilm Accumulation | | n | | % Biofilm Accumulation | n | | % Biofilm Accumulation | | | n | | % Biofilm Accumulation | n |
| Stationary planktonic phase | 88.9 ± 6.7 | 6 | | 74.9 ± 9.4 | | 7 | 29.9 ± 9.1 | | 6 | 18.4 ± 6.8 | | | 6 | 15.9 ± 3.0 | | | 6 | 89.8 ± 7.0 | | | 6 | 84.3 ± 3.0 | | 5 | | 72.5 ± 8.6 | 6 | | 63.3 ± 11.0 | | | 5 | | 54.7 ± 12.1 | 5 |
| BF | 115.4 ± 8.8 | 10 | | 85.2 ± 14.1 | | 8 | 42.8 ± 15.1 | | 10 | 32.8 ± 4.0 | | | 10 | 21.8 ± 3.2 | | | 9 | 66.3 ± 13.0 | | | 6 | 79.7 ± 22.4 | | 6 | | 61.4 ± 14.8 | 5 | | 50.6 ± 5.6 | | | 4 | | 58.1 ± 3.2 | 4 |
| ECM | 108.7 ± 14.8 | 8 | | 93.1 ± 30.8 | | 8 | 44.6 ± 16.2 | | 9 | 35.4 ± 8.5 | | | 7 | 28.7 ± 11.1 | | | 8 | 63.1 ± 12.6 | | | 6 | 73.7 ± 24.4 | | 6 | | 77.6 ± 8.6 | 4 | | 82.4 ± 9.9 | | | 4 | | 75.6 ± 17.4 | 4 |
| SP | 99.1 ± 6.2 | 10 | | 82.0 ± 8.5 | | 9 | 39.2 ± 12.1 | | 10 | 31.8 ± 7.2 | | | 10 | 24.7 ± 9.2 | | | 10 | 63.8 ± 5.8 | | | 5 | 81.2 ± 11.5 | | 5 | | 71.3 ± 0.8 | 4 | | 59.7 ± 2.5 | | | 4 | | 66.1 ± 11.6 | 5 |
| **Tobramycin Surface Dissociation Assay** | | | | | | | | | | | | | | | | | | | | | | | | | | | | | | | | | | | |
|  | **1.0 μM** | | | | **1.5 μM** | | | **2.0 μM** | | | | **3.0 μM** | | | | **20 μM** | | | | **40 μM** | | | | | **60 μM** | | | | | **100 μM** | | | **150 μM** | | |
| Samples | %Biofilm Remaining | | n | | %Biofilm Remaining | | n | %Biofilm Remaining | | | n | %Biofilm Remaining | | | n | %Biofilm Remaining | | | n | %Biofilm Remaining | | | n | | %Biofilm Remaining | | | n | | %Biofilm Remaining | n | | %Biofilm Remaining | | n |
| Stationary planktonic phase | 93.3 ± 5.3 | | 6 | | 97.9 ± 2.5 | | 5 | 49.7 ± 6.2 | | | 5 | 39.6 ± 12.6 | | | 6 | 43.3 ± 8.7 | | | 6 | 40.8 ± 3.9 | | | 5 | | 34.6 ± 4.5 | | | 5 | | 35.2 ± 6.7 | 5 | | 45.8 ± 14.8 | | 6 |
| BF | 92.1 ± 6.3 | | 6 | | 101.4 ± 6.8 | | 5 | 90.4 ± 1.1 | | | 5 | 81.9 ± 3.8 | | | 6 | 86.7 ± 1.3 | | | 5 | 75.1 ± 9.7 | | | 6 | | 68.3 ± 9.8 | | | 6 | | 68.9 ± 6.4 | 6 | | 65.1 ± 6.0 | | 6 |
| ECM | 100.5 ± 1.4 | | 6 | | 102.3 ± 4.1 | | 6 | 100.1 ± 5.3 | | | 6 | 95.4 ± 6.5 | | | 6 | 66.9 ± 6.4 | | | 6 | 62.1 ± 7.3 | | | 6 | | 52.2 ± 3.7 | | | 5 | | 57.4 ± 8.8 | 6 | | 51.0 ± 5.4 | | 5 |
| SP | 100.5 ± 1.2 | | 5 | | 99.1 ± 4.1 | | 5 | 95.6 ± 9.8 | | | 6 | 89.6 ± 12.7 | | | 6 | 55.6 ± 8.0 | | | 6 | 44.5 ± 5.5 | | | 6 | | 46.0 ± 3.9 | | | 5 | | 43.2 ± 5.7 | 6 | | 38.6 ± 6.8 | | 6 |
| **Colistin Surface Dissociation Assay** | | | | | | | | | | | | | | | | | | | | | | | | | | | | | | | | | | | |
|  | **0.25 μg/mL** | | | **0.5 μg/mL** | | | **1.0 μg/mL** | | | **2.0 μg/mL** | | | | **15 μg/mL** | | |  | **30 μg/mL** | | | | **50 μg/mL** | | | | **75 μg/mL** | | | **100 μg/mL** | | | | | **150 μg/mL** | |
| Samples | %Biofilm Remaining | n | | %Biofilm Remaining | | n | %Biofilm Remaining | | n | %Biofilm Remaining | | | n | %Biofilm Remaining | | | n | %Biofilm Remaining | | | n | %Biofilm Remaining | | n | | %Biofilm Remaining | n | | %Biofilm Remaining | | | n | | %Biofilm Remaining | n |
| Stationary planktonic phase | 89.7 ± 5.3 | 5 | | 88.4 ± 17.6 | | 5 | 62.4 ± 14.6 | | 6 | 50.4 ± 9.0 | | | 5 | 24.9 ± 3.4 | | | 5 | 27.1 ± 6.4 | | | 5 | 23.2 ± 5.6 | | 5 | | 20.0 ± 2.3 | 6 | | 21.4 ± 4.2 | | | 6 | | 22.5 ± 3.2 | 5 |
| BF | 94.8 ± 5.0 | 5 | | 99.4 ± 2.5 | | 5 | 102.1 ± 2.6 | | 5 | 97.8 ± 5.7 | | | 5 | 79.7 ± 8.6 | | | 6 | 66.8 ± 14.9 | | | 6 | 51.2 ± 13.9 | | 6 | | 53.4 ± 16.5 | 6 | | 54.6 ± 15.3 | | | 6 | | 48.4 ± 17.5 | 6 |
| ECM | 99.2 ± 2.6 | 5 | | 97.6 ± 1.7 | | 5 | 98.5 ± 4.9 | | 6 | 91.1 ± 6.4 | | | 6 | 80.5 ± 10.1 | | | 6 | 59.1 ± 12.8 | | | 6 | 38.0 ± 4.0 | | 5 | | 41.4 ± 8.7 | 6 | | 37.2 ± 9.1 | | | 6 | | 34.1 ± 3.3 | 5 |
| SP | 94.2 ± 2.7 | 5 | | 98.9 ± 9.3 | | 6 | 94.9 ± 11.8 | | 6 | 86.3 ± 9.9 | | | 5 | 77.3 ± 4.9 | | | 5 | 47.7 ± 4.7 | | | 5 | 35.3 ± 5.9 | | 6 | | 30.0 ± 3.8 | 6 | | 27.1 ± 8.1 | | | 6 | | 23.0 ± 5.3 | 6 |
| Normalized to untreated controls for each subpopulation. Stained with crystal violet and absorbance taken at 550 nm. Data given as mean of biological replicates ± SD, where there were four technical replicates of each biological replicate (n). | | | | | | | | | | | | | | | | | | | | | | | | | | | | | | | | | | | |
