# Supplementary material for: Characterization of Distinct Biofilm Cell Subpopulations and Implications in Quorum Sensing and Antibiotic Resistance
Source: mBio. 2022 Jun 13;13(3):e00191-22. doi: 10.1128/mbio.00191-22 (PMC9239111; doi:10.1128/mbio.00191-22)
Supplement: TABLE S3 [file mbio.00191-22-s0003.docx]

| Table S3: Nomenclature of discussed molecule abbreviations with structures | | |
| --- | --- | --- |
| Nomenclature | **Abbreviation** | **Structure** |
| N-butanoyl-L-homoserine lactone | C_4_-HSL |  |
| N-3-oxo-dodecanoyl-L-homoserine lactone | N-3-oxo-C_12_-HSL |  |
| Pyocyanin | PYO |  |
| Phenazine-1-carboxamide | PCN |  |
| 2-heptyl-4-hydroxyquinoline | HHQ |  |
| 2-heptyl-3-hydroxy-4(1H)-quinolone | PQS |  |
| 2-heptyl-4-hydroxyquinoline-N-oxide | HQNO |  |
| 2-nonenyl-4-hydroxyquinoline | C_9:1_ NHQ |  |
| 2-nonyl-4-hydroxyquinoline | NHQ |  |
| 2-nonenyl-3-hydroxy-4(1H)-quinolone | C_9:1_ PQS |  |
| 2-nonenyl-4-hydroxyquinoline-N-oxide | C_9:1_ HQNO |  |
| 2-nonyl-3-hydroxy-4(1H)-quinolone | C_9_ PQS |  |
| 2-nonyl-4-hydroxyquinoline-N-oxide | C_9_ HQNO |  |
| 2-undecenyl-4-hydroxyquinoline | C_11:1_ UHQ |  |
| α−L-rhamnopyranosyl-β-hydroxydecanoate | Rha-C_10_ |  |
| α−L-rhamnopyranosyl−β−hydroxydodecanoate | Rha-C_12_ |  |
| α−L-rhamnopyranosyl−α−L-hydroxyoctanoyl−β−hydroxydecanoate | Rha-C_8_-C_10_ |  |
| α−L−rhamnopyranosyl−α−L-rhamnopyranosyl−β−hydroxydecanoate | Rha-Rha-C_10_ |  |
| α−L-rhamnopyranosyl−α−L-hydroxydecanoyl−β−hydroxydecanoate | Rha-C_10_-C_10_ |  |
| α−L−rhamnopyranosyl−α−L−β−hydroxydecanoyl−β-hydroxydodecenoate | Rha-C_10_-C_12:1_ |  |
| α−L−rhamnopyranosyl−α−L−β−hydroxydecanoyl−β−dodecanoate | Rha-C_10_-C_12_ |  |
| α−L−rhamnopyranosyl−α−L−rhamnopyranosyl−β−hydroxyoctanoyl−β−hydroxydecanoate | Rha-Rha-C_8_-C_10_ |  |
| α−L−rhamnopyranosyl−α−L−rhamnopyranosyl−β−hydroxydecanoyl−β-hydroxydecanoate | Rha-Rha-C_10_-C_10_ |  |
| Cyclic guanosine monophosphate | Cyclic-GMP |  |
| Bis-(3',5')-cyclic dimeric guanosine monophosphate | Cyclic-di-GMP |  |
