# Supplementary material for: Characterization of Distinct Biofilm Cell Subpopulations and Implications in Quorum Sensing and Antibiotic Resistance
Source: mBio. 2022 Jun 13;13(3):e00191-22. doi: 10.1128/mbio.00191-22 (PMC9239111; doi:10.1128/mbio.00191-22)
Supplement: TABLE S4 [file mbio.00191-22-s0004.docx]

| **Table S4: Calculated Pearson’s coefficients for all ions of interest**. Blue boxes indicate Pearson’s coefficient value >0.7 and green boxes indicate Pearson’s coefficient value >0.9. Black represents an ion being compared against itself. | | | | | | | | | | | | | | | | | | | | | | | | | | | | | | | | | | | | | | | | | | | | | |
| --- | --- | --- | --- | --- | --- | --- | --- | --- | --- | --- | --- | --- | --- | --- | --- | --- | --- | --- | --- | --- | --- | --- | --- | --- | --- | --- | --- | --- | --- | --- | --- | --- | --- | --- | --- | --- | --- | --- | --- | --- | --- | --- | --- | --- | --- |
|  | ***m/z*** | 172 | 368 | 320 | 224 | 233 | 246 | 247 | 266 | 282 | 292 | 294 | 308 | 310 | 320 | 357 | 363 | 385 | 477 | 481 | 499 | 503 | 505 | 527 | 531 | 533 | 553 | 645 | 673 | 346 | 691 | 293 | 335 | 329 | 356 | 379 | 383 | 404 | 463 | 616 | 702 | 394 | 430 | 466 | 482 |
| C4-HSL (+H) | 172 | 1 | 0.432 | 0.108 | 0.423 | 0.453 | 0.626 | 0.6 | 0.098 | 0.092 | 0.085 | 0.082 | 0.084 | 0.082 | 0.108 | 0.602 | 0.479 | 0.524 | 0.375 | 0.344 | 0.38 | 0.384 | 0.348 | 0.356 | 0.324 | 0.225 | 0.168 | 0.174 | 0.196 | 0.409 | 0.169 | 0.171 | 0.182 | 0.534 | 0.573 | 0.5 | 0.518 | 0.418 | 0.387 | 0.216 | 0.191 | 0.407 | 0.42 | 0.381 | 0.328 |
| C4-HSL (+Au) | 368 | 0.432 | 1 | 0.395 | 0.672 | 0.578 | 0.635 | 0.614 | 0.417 | 0.44 | 0.376 | 0.386 | 0.436 | 0.44 | 0.395 | 0.627 | 0.805 | 0.73 | 0.797 | 0.803 | 0.664 | 0.732 | 0.788 | 0.709 | 0.756 | 0.663 | 0.515 | 0.614 | 0.644 | 0.675 | 0.689 | 0.45 | 0.566 | 0.671 | 0.642 | 0.679 | 0.711 | 0.614 | 0.629 | 0.642 | 0.648 | 0.803 | 0.736 | 0.676 | 0.777 |
| N-(3-Oxo-C12)-HSL (+Na) | 320 | 0.108 | 0.395 | 1 | 0.392 | 0.138 | 0.316 | 0.118 | 0.944 | 0.927 | 0.968 | 0.974 | 0.932 | 0.933 | 0 | 0.277 | 0.428 | 0.362 | 0.418 | 0.431 | 0.44 | 0.41 | 0.487 | 0.5 | 0.466 | 0.749 | 0.775 | 0.682 | 0.555 | 0.472 | 0.51 | 0.911 | 0.888 | 0.252 | 0.272 | 0.354 | 0.328 | 0.334 | 0.305 | 0.641 | 0.476 | 0.413 | 0.324 | 0.354 | 0.441 |
| Phenazine-1-Carboxamide (+H) | 224 | 0.423 | 0.672 | 0.392 | 1 | 0.539 | 0.605 | 0.586 | 0.398 | 0.402 | 0.374 | 0.382 | 0.399 | 0.399 | 0.392 | 0.53 | 0.68 | 0.637 | 0.608 | 0.597 | 0.525 | 0.559 | 0.611 | 0.585 | 0.55 | 0.515 | 0.452 | 0.53 | 0.543 | 0.582 | 0.547 | 0.437 | 0.506 | 0.615 | 0.544 | 0.586 | 0.629 | 0.481 | 0.494 | 0.522 | 0.55 | 0.656 | 0.574 | 0.53 | 0.569 |
| Pyocyanin (+Na) | 233 | 0.453 | 0.578 | 0.138 | 0.539 | 1 | 0.539 | 0.562 | 0.137 | 0.158 | 0.107 | 0.115 | 0.144 | 0.156 | 0.138 | 0.466 | 0.532 | 0.513 | 0.468 | 0.48 | 0.361 | 0.403 | 0.434 | 0.396 | 0.404 | 0.31 | 0.194 | 0.272 | 0.316 | 0.518 | 0.384 | 0.182 | 0.254 | 0.544 | 0.488 | 0.506 | 0.521 | 0.442 | 0.408 | 0.284 | 0.349 | 0.542 | 0.531 | 0.423 | 0.453 |
| Phenazine-1-Carboxamide (+Na) | 246 | 0.626 | 0.635 | 0.316 | 0.605 | 0.539 | 1 | 0.702 | 0.33 | 0.324 | 0.285 | 0.296 | 0.307 | 0.305 | 0.316 | 0.702 | 0.676 | 0.689 | 0.615 | 0.581 | 0.577 | 0.595 | 0.592 | 0.58 | 0.599 | 0.483 | 0.402 | 0.433 | 0.456 | 0.581 | 0.446 | 0.374 | 0.419 | 0.653 | 0.681 | 0.651 | 0.677 | 0.572 | 0.568 | 0.474 | 0.451 | 0.613 | 0.601 | 0.571 | 0.552 |
| Phenazine-1-Carboxamide (+Na + H) | 247 | 0.6 | 0.614 | 0.118 | 0.586 | 0.562 | 0.702 | 1 | 0.118 | 0.114 | 0.085 | 0.085 | 0.1 | 0.106 | 0.118 | 0.66 | 0.646 | 0.666 | 0.572 | 0.532 | 0.512 | 0.55 | 0.533 | 0.509 | 0.494 | 0.324 | 0.239 | 0.33 | 0.389 | 0.525 | 0.389 | 0.187 | 0.245 | 0.66 | 0.652 | 0.624 | 0.665 | 0.526 | 0.533 | 0.37 | 0.413 | 0.591 | 0.576 | 0.535 | 0.494 |
| HHQ (+Na) | 266 | 0.098 | 0.417 | 0.944 | 0.398 | 0.137 | 0.33 | 0.118 | 1 | 0.965 | 0.95 | 0.965 | 0.948 | 0.933 | 0.944 | 0.283 | 0.454 | 0.361 | 0.464 | 0.482 | 0.464 | 0.453 | 0.539 | 0.519 | 0.519 | 0.802 | 0.768 | 0.659 | 0.543 | 0.434 | 0.511 | 0.936 | 0.889 | 0.257 | 0.278 | 0.339 | 0.32 | 0.319 | 0.31 | 0.649 | 0.473 | 0.446 | 0.344 | 0.371 | 0.502 |
| PQS (+Na) or HQNO (+Na) | 282 | 0.092 | 0.44 | 0.927 | 0.402 | 0.158 | 0.324 | 0.114 | 0.965 | 1 | 0.93 | 0.956 | 0.98 | 0.974 | 0.927 | 0.261 | 0.464 | 0.349 | 0.473 | 0.502 | 0.445 | 0.445 | 0.546 | 0.513 | 0.525 | 0.806 | 0.73 | 0.643 | 0.536 | 0.453 | 0.523 | 0.908 | 0.927 | 0.255 | 0.259 | 0.33 | 0.307 | 0.322 | 0.314 | 0.635 | 0.471 | 0.469 | 0.362 | 0.372 | 0.523 |
| C9:1 NHQ (+Na) | 292 | 0.085 | 0.376 | 0.968 | 0.374 | 0.107 | 0.285 | 0.085 | 0.95 | 0.93 | 1 | 0.976 | 0.945 | 0.931 | 0.968 | 0.248 | 0.405 | 0.339 | 0.404 | 0.419 | 0.443 | 0.399 | 0.481 | 0.501 | 0.46 | 0.757 | 0.796 | 0.689 | 0.553 | 0.442 | 0.494 | 0.937 | 0.879 | 0.231 | 0.246 | 0.329 | 0.303 | 0.305 | 0.284 | 0.647 | 0.469 | 0.388 | 0.297 | 0.343 | 0.436 |
| NHQ (+Na) | 294 | 0.082 | 0.386 | 0.974 | 0.382 | 0.115 | 0.296 | 0.085 | 0.965 | 0.956 | 0.976 | 1 | 0.962 | 0.952 | 0.974 | 0.246 | 0.414 | 0.331 | 0.417 | 0.435 | 0.43 | 0.405 | 0.493 | 0.494 | 0.473 | 0.774 | 0.781 | 0.676 | 0.545 | 0.44 | 0.505 | 0.926 | 0.897 | 0.226 | 0.242 | 0.316 | 0.293 | 0.298 | 0.279 | 0.642 | 0.465 | 0.405 | 0.307 | 0.335 | 0.454 |
| C9:1 PQS (+Na) or C9:1 HQNO (+Na) | 308 | 0.084 | 0.436 | 0.932 | 0.399 | 0.144 | 0.307 | 0.1 | 0.948 | 0.98 | 0.945 | 0.962 | 1 | 0.977 | 0.932 | 0.253 | 0.456 | 0.347 | 0.466 | 0.493 | 0.453 | 0.44 | 0.542 | 0.525 | 0.521 | 0.814 | 0.773 | 0.678 | 0.556 | 0.463 | 0.533 | 0.897 | 0.936 | 0.244 | 0.252 | 0.331 | 0.306 | 0.325 | 0.315 | 0.658 | 0.482 | 0.455 | 0.355 | 0.371 | 0.514 |
| C9 PQS (+Na) or C9 HQNO (+Na) | 310 | 0.082 | 0.44 | 0.933 | 0.399 | 0.156 | 0.305 | 0.106 | 0.933 | 0.974 | 0.931 | 0.952 | 0.977 | 1 | 0.933 | 0.248 | 0.456 | 0.345 | 0.461 | 0.492 | 0.434 | 0.43 | 0.532 | 0.507 | 0.509 | 0.794 | 0.733 | 0.663 | 0.552 | 0.472 | 0.541 | 0.878 | 0.931 | 0.248 | 0.248 | 0.327 | 0.305 | 0.321 | 0.307 | 0.638 | 0.48 | 0.465 | 0.354 | 0.362 | 0.511 |
| C11:1 UHQ (+Na) | 320 | 0.108 | 0.395 | 0 | 0.392 | 0.138 | 0.316 | 0.118 | 0.944 | 0.927 | 0.968 | 0.974 | 0.932 | 0.933 | 1 | 0.277 | 0.428 | 0.362 | 0.418 | 0.431 | 0.44 | 0.41 | 0.487 | 0.5 | 0.466 | 0.749 | 0.775 | 0.682 | 0.555 | 0.472 | 0.51 | 0.911 | 0.888 | 0.252 | 0.272 | 0.354 | 0.328 | 0.334 | 0.305 | 0.641 | 0.476 | 0.413 | 0.324 | 0.354 | 0.441 |
| Rha-C10 (+Na) | 357 | 0.602 | 0.627 | 0.277 | 0.53 | 0.466 | 0.702 | 0.66 | 0.283 | 0.261 | 0.248 | 0.246 | 0.253 | 0.248 | 0.277 | 1 | 0.691 | 0.754 | 0.674 | 0.629 | 0.709 | 0.705 | 0.652 | 0.666 | 0.642 | 0.512 | 0.453 | 0.485 | 0.519 | 0.597 | 0.491 | 0.333 | 0.373 | 0.677 | 0.946 | 0.714 | 0.736 | 0.675 | 0.673 | 0.549 | 0.503 | 0.608 | 0.668 | 0.676 | 0.601 |
| Rha-C12 (+H) | 363 | 0.479 | 0.805 | 0.428 | 0.68 | 0.532 | 0.676 | 0.646 | 0.454 | 0.464 | 0.405 | 0.414 | 0.456 | 0.456 | 0.428 | 0.691 | 1 | 0.777 | 0.832 | 0.807 | 0.717 | 0.791 | 0.828 | 0.751 | 0.79 | 0.679 | 0.54 | 0.633 | 0.663 | 0.665 | 0.669 | 0.498 | 0.594 | 0.696 | 0.69 | 0.72 | 0.753 | 0.641 | 0.669 | 0.679 | 0.658 | 0.809 | 0.734 | 0.712 | 0.774 |
| Rha-C12 (+Na) | 385 | 0.524 | 0.73 | 0.362 | 0.637 | 0.513 | 0.689 | 0.666 | 0.361 | 0.349 | 0.339 | 0.331 | 0.347 | 0.345 | 0.362 | 0.754 | 0.777 | 1 | 0.76 | 0.727 | 0.769 | 0.764 | 0.748 | 0.771 | 0.727 | 0.598 | 0.547 | 0.622 | 0.658 | 0.697 | 0.629 | 0.419 | 0.476 | 0.734 | 0.753 | 0.775 | 0.852 | 0.721 | 0.738 | 0.666 | 0.644 | 0.705 | 0.742 | 0.761 | 0.684 |
| Rha-C8-C10 (+H) | 477 | 0.375 | 0.797 | 0.418 | 0.608 | 0.468 | 0.615 | 0.572 | 0.464 | 0.473 | 0.404 | 0.417 | 0.466 | 0.461 | 0.418 | 0.674 | 0.832 | 0.76 | 1 | 0.87 | 0.779 | 0.854 | 0.887 | 0.8 | 0.874 | 0.754 | 0.595 | 0.694 | 0.726 | 0.644 | 0.745 | 0.487 | 0.59 | 0.655 | 0.676 | 0.7 | 0.728 | 0.659 | 0.706 | 0.753 | 0.719 | 0.805 | 0.757 | 0.755 | 0.844 |
| Rha-Rha-C10 (+H) | 481 | 0.344 | 0.803 | 0.431 | 0.597 | 0.48 | 0.581 | 0.532 | 0.482 | 0.502 | 0.419 | 0.435 | 0.493 | 0.492 | 0.431 | 0.629 | 0.807 | 0.727 | 0.87 | 1 | 0.753 | 0.821 | 0.869 | 0.776 | 0.861 | 0.76 | 0.588 | 0.684 | 0.717 | 0.654 | 0.771 | 0.498 | 0.604 | 0.63 | 0.641 | 0.663 | 0.69 | 0.641 | 0.674 | 0.736 | 0.708 | 0.813 | 0.773 | 0.74 | 0.921 |
| Rha-C8-C10 (+Na) | 499 | 0.38 | 0.664 | 0.44 | 0.525 | 0.361 | 0.577 | 0.512 | 0.464 | 0.445 | 0.443 | 0.43 | 0.453 | 0.434 | 0.44 | 0.709 | 0.717 | 0.769 | 0.779 | 0.753 | 1 | 0.826 | 0.791 | 0.855 | 0.794 | 0.709 | 0.701 | 0.749 | 0.769 | 0.665 | 0.702 | 0.497 | 0.537 | 0.65 | 0.715 | 0.732 | 0.736 | 0.743 | 0.776 | 0.801 | 0.726 | 0.652 | 0.739 | 0.819 | 0.732 |
| Rha-Rha-C10 (+Na) | 503 | 0.384 | 0.732 | 0.41 | 0.559 | 0.403 | 0.595 | 0.55 | 0.453 | 0.445 | 0.399 | 0.405 | 0.44 | 0.43 | 0.41 | 0.705 | 0.791 | 0.764 | 0.854 | 0.821 | 0.826 | 1 | 0.873 | 0.814 | 0.854 | 0.726 | 0.614 | 0.702 | 0.738 | 0.62 | 0.714 | 0.479 | 0.553 | 0.655 | 0.705 | 0.69 | 0.722 | 0.664 | 0.709 | 0.772 | 0.714 | 0.741 | 0.734 | 0.764 | 0.805 |
| Rha-C10-C10 (+H) | 505 | 0.348 | 0.788 | 0.487 | 0.611 | 0.434 | 0.592 | 0.533 | 0.539 | 0.546 | 0.481 | 0.493 | 0.542 | 0.532 | 0.487 | 0.652 | 0.828 | 0.748 | 0.887 | 0.869 | 0.791 | 0.873 | 1 | 0.816 | 0.886 | 0.801 | 0.652 | 0.745 | 0.763 | 0.637 | 0.765 | 0.552 | 0.649 | 0.639 | 0.656 | 0.674 | 0.707 | 0.631 | 0.679 | 0.798 | 0.747 | 0.801 | 0.736 | 0.744 | 0.852 |
| Rha-C10-C10 (+Na) | 527 | 0.356 | 0.709 | 0.5 | 0.585 | 0.396 | 0.58 | 0.509 | 0.519 | 0.513 | 0.501 | 0.494 | 0.525 | 0.507 | 0.5 | 0.666 | 0.751 | 0.771 | 0.8 | 0.776 | 0.855 | 0.814 | 0.816 | 1 | 0.81 | 0.756 | 0.74 | 0.794 | 0.802 | 0.704 | 0.747 | 0.545 | 0.613 | 0.657 | 0.676 | 0.745 | 0.745 | 0.736 | 0.777 | 0.83 | 0.766 | 0.692 | 0.746 | 0.816 | 0.742 |
| Rha-C10-C12:1 (+H) | 531 | 0.324 | 0.756 | 0.466 | 0.55 | 0.404 | 0.559 | 0.494 | 0.519 | 0.525 | 0.46 | 0.473 | 0.521 | 0.509 | 0.466 | 0.642 | 0.79 | 0.727 | 0.874 | 0.861 | 0.794 | 0.854 | 0.886 | 0.81 | 1 | 0.81 | 0.635 | 0.723 | 0.741 | 0.632 | 0.744 | 0.528 | 0.62 | 0.61 | 0.646 | 0.676 | 0.686 | 0.652 | 0.699 | 0.788 | 0.717 | 0.767 | 0.734 | 0.76 | 0.842 |
| Rha-C10-C12 (+H) | 533 | 0.225 | 0.663 | 0.749 | 0.515 | 0.31 | 0.483 | 0.324 | 0.802 | 0.806 | 0.757 | 0.774 | 0.814 | 0.794 | 0.749 | 0.512 | 0.679 | 0.598 | 0.754 | 0.76 | 0.709 | 0.726 | 0.801 | 0.756 | 0.81 | 1 | 0.836 | 0.801 | 0.731 | 0.605 | 0.731 | 0.75 | 0.832 | 0.466 | 0.509 | 0.572 | 0.56 | 0.562 | 0.582 | 0.83 | 0.675 | 0.666 | 0.613 | 0.634 | 0.762 |
| Rha-C10-C12:1 (+Na) | 553 | 0.168 | 0.515 | 0.775 | 0.452 | 0.194 | 0.402 | 0.239 | 0.768 | 0.73 | 0.796 | 0.781 | 0.773 | 0.733 | 0.775 | 0.453 | 0.54 | 0.547 | 0.595 | 0.588 | 0.701 | 0.614 | 0.652 | 0.74 | 0.653 | 0.836 | 1 | 0.854 | 0.756 | 0.593 | 0.671 | 0.728 | 0.767 | 0.39 | 0.455 | 0.546 | 0.515 | 0.557 | 0.563 | 0.832 | 0.671 | 0.492 | 0.515 | 0.604 | 0.583 |
| Rha-Rha-C8-C10 (+Na) | 645 | 0.174 | 0.614 | 0.682 | 0.53 | 0.272 | 0.433 | 0.33 | 0.659 | 0.643 | 0.689 | 0.676 | 0.678 | 0.663 | 0.682 | 0.485 | 0.633 | 0.622 | 0.694 | 0.684 | 0.749 | 0.702 | 0.745 | 0.794 | 0.723 | 0.801 | 0.854 | 1 | 0.862 | 0.633 | 0.78 | 0.646 | 0.718 | 0.49 | 0.501 | 0.578 | 0.586 | 0.57 | 0.599 | 0.875 | 0.797 | 0.606 | 0.591 | 0.658 | 0.674 |
| Rha-Rha-C10-C10 (+Na) | 673 | 0.196 | 0.644 | 0.555 | 0.543 | 0.316 | 0.456 | 0.389 | 0.543 | 0.536 | 0.553 | 0.545 | 0.556 | 0.552 | 0.555 | 0.519 | 0.663 | 0.658 | 0.726 | 0.717 | 0.769 | 0.738 | 0.763 | 0.802 | 0.741 | 0.731 | 0.756 | 0.862 | 1 | 0.635 | 0.787 | 0.543 | 0.627 | 0.542 | 0.543 | 0.603 | 0.62 | 0.598 | 0.641 | 0.838 | 0.818 | 0.642 | 0.645 | 0.702 | 0.701 |
| Cyclic-GMP (+H) | 346 | 0.409 | 0.675 | 0.472 | 0.582 | 0.518 | 0.581 | 0.525 | 0.434 | 0.453 | 0.442 | 0.44 | 0.463 | 0.472 | 0.472 | 0.597 | 0.665 | 0.697 | 0.644 | 0.654 | 0.665 | 0.62 | 0.637 | 0.704 | 0.632 | 0.605 | 0.593 | 0.633 | 0.635 | 1 | 0.647 | 0.475 | 0.548 | 0.628 | 0.617 | 0.705 | 0.69 | 0.745 | 0.707 | 0.631 | 0.615 | 0.637 | 0.74 | 0.707 | 0.608 |
| Cyclic-di-GMP (+H) | 691 | 0.169 | 0.689 | 0.51 | 0.547 | 0.384 | 0.446 | 0.389 | 0.511 | 0.523 | 0.494 | 0.505 | 0.533 | 0.541 | 0.51 | 0.491 | 0.669 | 0.629 | 0.745 | 0.771 | 0.702 | 0.714 | 0.765 | 0.747 | 0.744 | 0.731 | 0.671 | 0.78 | 0.787 | 0.647 | 1 | 0.5 | 0.606 | 0.524 | 0.514 | 0.564 | 0.596 | 0.588 | 0.605 | 0.768 | 0.767 | 0.691 | 0.705 | 0.665 | 0.758 |
| **BF UNIQUE** | 293 | 0.171 | 0.45 | 0.911 | 0.437 | 0.182 | 0.374 | 0.187 | 0.936 | 0.908 | 0.937 | 0.926 | 0.897 | 0.878 | 0.911 | 0.333 | 0.498 | 0.419 | 0.487 | 0.498 | 0.497 | 0.479 | 0.552 | 0.545 | 0.528 | 0.75 | 0.728 | 0.646 | 0.543 | 0.475 | 0.5 | 1 | 0.851 | 0.326 | 0.33 | 0.399 | 0.382 | 0.367 | 0.357 | 0.64 | 0.482 | 0.475 | 0.383 | 0.419 | 0.507 |
|  | 335 | 0.182 | 0.566 | 0.888 | 0.506 | 0.254 | 0.419 | 0.245 | 0.889 | 0.927 | 0.879 | 0.897 | 0.936 | 0.931 | 0.888 | 0.373 | 0.594 | 0.476 | 0.59 | 0.604 | 0.537 | 0.553 | 0.649 | 0.613 | 0.62 | 0.832 | 0.767 | 0.718 | 0.627 | 0.548 | 0.606 | 0.851 | 1 | 0.372 | 0.373 | 0.45 | 0.438 | 0.42 | 0.419 | 0.707 | 0.568 | 0.585 | 0.465 | 0.475 | 0.61 |
| **SP UNIQUE** | 329 | 0.534 | 0.671 | 0.252 | 0.615 | 0.544 | 0.653 | 0.66 | 0.257 | 0.255 | 0.231 | 0.226 | 0.244 | 0.248 | 0.252 | 0.677 | 0.696 | 0.734 | 0.655 | 0.63 | 0.65 | 0.655 | 0.639 | 0.657 | 0.61 | 0.466 | 0.39 | 0.49 | 0.542 | 0.628 | 0.524 | 0.326 | 0.372 | 1 | 0.689 | 0.688 | 0.724 | 0.627 | 0.635 | 0.528 | 0.548 | 0.647 | 0.675 | 0.66 | 0.596 |
|  | 356 | 0.573 | 0.642 | 0.272 | 0.544 | 0.488 | 0.681 | 0.652 | 0.278 | 0.259 | 0.246 | 0.242 | 0.252 | 0.248 | 0.272 | 0.946 | 0.69 | 0.753 | 0.676 | 0.641 | 0.715 | 0.705 | 0.656 | 0.676 | 0.646 | 0.509 | 0.455 | 0.501 | 0.543 | 0.617 | 0.514 | 0.33 | 0.373 | 0.689 | 1 | 0.714 | 0.735 | 0.68 | 0.676 | 0.561 | 0.528 | 0.617 | 0.682 | 0.689 | 0.615 |
|  | 379 | 0.5 | 0.679 | 0.354 | 0.586 | 0.506 | 0.651 | 0.624 | 0.339 | 0.33 | 0.329 | 0.316 | 0.331 | 0.327 | 0.354 | 0.714 | 0.72 | 0.775 | 0.7 | 0.663 | 0.732 | 0.69 | 0.674 | 0.745 | 0.676 | 0.572 | 0.546 | 0.578 | 0.603 | 0.705 | 0.564 | 0.399 | 0.45 | 0.688 | 0.714 | 1 | 0.786 | 0.768 | 0.778 | 0.628 | 0.592 | 0.636 | 0.714 | 0.768 | 0.6 |
|  | 383 | 0.518 | 0.711 | 0.328 | 0.629 | 0.521 | 0.677 | 0.665 | 0.32 | 0.307 | 0.303 | 0.293 | 0.306 | 0.305 | 0.328 | 0.736 | 0.753 | 0.852 | 0.728 | 0.69 | 0.736 | 0.722 | 0.707 | 0.745 | 0.686 | 0.56 | 0.515 | 0.586 | 0.62 | 0.69 | 0.596 | 0.382 | 0.438 | 0.724 | 0.735 | 0.786 | 1 | 0.727 | 0.744 | 0.628 | 0.618 | 0.676 | 0.721 | 0.749 | 0.634 |
|  | 404 | 0.418 | 0.614 | 0.334 | 0.481 | 0.442 | 0.572 | 0.526 | 0.319 | 0.322 | 0.305 | 0.298 | 0.325 | 0.321 | 0.334 | 0.675 | 0.641 | 0.721 | 0.659 | 0.641 | 0.743 | 0.664 | 0.631 | 0.736 | 0.652 | 0.562 | 0.557 | 0.57 | 0.598 | 0.745 | 0.588 | 0.367 | 0.42 | 0.627 | 0.68 | 0.768 | 0.727 | 1 | 0.826 | 0.62 | 0.585 | 0.577 | 0.761 | 0.793 | 0.577 |
|  | 463 | 0.387 | 0.629 | 0.305 | 0.494 | 0.408 | 0.586 | 0.533 | 0.31 | 0.314 | 0.284 | 0.279 | 0.315 | 0.307 | 0.305 | 0.673 | 0.669 | 0.738 | 0.706 | 0.674 | 0.776 | 0.709 | 0.679 | 0.777 | 0.699 | 0.582 | 0.563 | 0.599 | 0.641 | 0.707 | 0.605 | 0.357 | 0.419 | 0.635 | 0.676 | 0.778 | 0.744 | 0.826 | 1 | 0.661 | 0.634 | 0.594 | 0.748 | 0.826 | 0.607 |
|  | 616 | 0.216 | 0.642 | 0.641 | 0.522 | 0.284 | 0.474 | 0.37 | 0.649 | 0.635 | 0.647 | 0.642 | 0.658 | 0.638 | 0.641 | 0.549 | 0.679 | 0.666 | 0.753 | 0.736 | 0.801 | 0.772 | 0.798 | 0.83 | 0.788 | 0.83 | 0.832 | 0.875 | 0.838 | 0.631 | 0.768 | 0.64 | 0.707 | 0.528 | 0.561 | 0.628 | 0.628 | 0.62 | 0.661 | 1 | 0.782 | 0.638 | 0.64 | 0.716 | 0.722 |
|  | 702 | 0.191 | 0.648 | 0.476 | 0.55 | 0.349 | 0.451 | 0.413 | 0.473 | 0.471 | 0.469 | 0.465 | 0.482 | 0.48 | 0.476 | 0.503 | 0.658 | 0.644 | 0.719 | 0.708 | 0.726 | 0.714 | 0.747 | 0.766 | 0.717 | 0.675 | 0.671 | 0.797 | 0.818 | 0.615 | 0.767 | 0.482 | 0.568 | 0.548 | 0.528 | 0.592 | 0.618 | 0.585 | 0.634 | 0.782 | 1 | 0.649 | 0.648 | 0.686 | 0.683 |
| **ECM UNIQUE** | 394 | 0.407 | 0.803 | 0.413 | 0.656 | 0.542 | 0.613 | 0.591 | 0.446 | 0.469 | 0.388 | 0.405 | 0.455 | 0.465 | 0.413 | 0.608 | 0.809 | 0.705 | 0.805 | 0.813 | 0.652 | 0.741 | 0.801 | 0.692 | 0.767 | 0.666 | 0.492 | 0.606 | 0.642 | 0.637 | 0.691 | 0.475 | 0.585 | 0.647 | 0.617 | 0.636 | 0.676 | 0.577 | 0.594 | 0.638 | 0.649 | 1 | 0.737 | 0.652 | 0.802 |
|  | 430 | 0.42 | 0.736 | 0.324 | 0.574 | 0.531 | 0.601 | 0.576 | 0.344 | 0.362 | 0.297 | 0.307 | 0.355 | 0.354 | 0.324 | 0.668 | 0.734 | 0.742 | 0.757 | 0.773 | 0.739 | 0.734 | 0.736 | 0.746 | 0.734 | 0.613 | 0.515 | 0.591 | 0.645 | 0.74 | 0.705 | 0.383 | 0.465 | 0.675 | 0.682 | 0.714 | 0.721 | 0.761 | 0.748 | 0.64 | 0.648 | 0.737 | 1 | 0.767 | 0.741 |
|  | 466 | 0.381 | 0.676 | 0.354 | 0.53 | 0.423 | 0.571 | 0.535 | 0.371 | 0.372 | 0.343 | 0.335 | 0.371 | 0.362 | 0.354 | 0.676 | 0.712 | 0.761 | 0.755 | 0.74 | 0.819 | 0.764 | 0.744 | 0.816 | 0.76 | 0.634 | 0.604 | 0.658 | 0.702 | 0.707 | 0.665 | 0.419 | 0.475 | 0.66 | 0.689 | 0.768 | 0.749 | 0.793 | 0.826 | 0.716 | 0.686 | 0.652 | 0.767 | 1 | 0.69 |
|  | 482 | 0.328 | 0.777 | 0.441 | 0.569 | 0.453 | 0.552 | 0.494 | 0.502 | 0.523 | 0.436 | 0.454 | 0.514 | 0.511 | 0.441 | 0.601 | 0.774 | 0.684 | 0.844 | 0.921 | 0.732 | 0.805 | 0.852 | 0.742 | 0.842 | 0.762 | 0.583 | 0.674 | 0.701 | 0.608 | 0.758 | 0.507 | 0.61 | 0.596 | 0.615 | 0.6 | 0.634 | 0.577 | 0.607 | 0.722 | 0.683 | 0.802 | 0.741 | 0.69 | 1 |
